# Selection, optimization and validation of ten chronic disease polygenic risk scores for clinical implementation in diverse US populations

**DOI:** 10.1038/s41591-024-02796-z

**Published:** 2024-02-19

**Authors:** Niall J. Lennon, Leah C. Kottyan, Christopher Kachulis, Noura S. Abul-Husn, Josh Arias, Gillian Belbin, Jennifer E. Below, Sonja I. Berndt, Wendy K. Chung, James J. Cimino, Ellen Wright Clayton, John J. Connolly, David R. Crosslin, Ozan Dikilitas, Digna R. Velez Edwards, QiPing Feng, Marissa Fisher, Robert R. Freimuth, Tian Ge, The GIANT Consortium, Sonja Berndt, Joel Hirschhorn, Ruth Loos, The All of Us Research Program, Joseph T. Glessner, Adam S. Gordon, Candace Patterson, Hakon Hakonarson, Maegan Harden, Margaret Harr, Joel N. Hirschhorn, Clive Hoggart, Li Hsu, Marguerite R. Irvin, Gail P. Jarvik, Elizabeth W. Karlson, Atlas Khan, Amit Khera, Krzysztof Kiryluk, Iftikhar Kullo, Katie Larkin, Nita Limdi, Jodell E. Linder, Ruth J. F. Loos, Yuan Luo, Edyta Malolepsza, Teri A. Manolio, Lisa J. Martin, Li McCarthy, Elizabeth M. McNally, James B. Meigs, Tesfaye B. Mersha, Jonathan D. Mosley, Anjene Musick, Bahram Namjou, Nihal Pai, Lorenzo L. Pesce, Ulrike Peters, Josh F. Peterson, Cynthia A. Prows, Megan J. Puckelwartz, Heidi L. Rehm, Dan M. Roden, Elisabeth A. Rosenthal, Robb Rowley, Konrad Teodor Sawicki, Daniel J. Schaid, Roelof A. J. Smit, Johanna L. Smith, Jordan W. Smoller, Minta Thomas, Hemant Tiwari, Diana M. Toledo, Nataraja Sarma Vaitinadin, David Veenstra, Theresa L. Walunas, Zhe Wang, Wei-Qi Wei, Chunhua Weng, Georgia L. Wiesner, Xianyong Yin, Eimear E. Kenny

**Affiliations:** 1 Broad Institute of MIT and Harvard, Cambridge, MA USA; 2 Cincinnati Children’s Hospital Medical Center, University of Cincinnati, Cincinnati, OH USA; 3 Icahn School of Medicine at Mount Sinai, New York, NY USA; 4 National Human Genome Research Institute, National Institutes of Health, Bethesda, MD USA; 5 Vanderbilt University Medical Center, Nashville, TN USA; 6 Columbia University, New York, NY USA; 7 University of Alabama at Birmingham, Birmingham, AL USA; 8 Children’s Hospital of Philadelphia, Philadelphia, PA USA; 9 Tulane University, New Orleans, LA USA; 10 University of Washington, Seattle, WA USA; 11 Mayo Clinic, Rochester, MI USA; 12 Mass General Brigham, Boston, MA USA; 13 Northwestern University, Evanston, IL USA; 14 Boston Children’s Hospital, Boston, MA USA; 15 Fred Hutchinson Cancer Center, Seattle, WA USA; 16 Novo Nordisk Foundation Center for Basic Metabolic Research, Faculty of Health and Medical Sciences, University of Copenhagen, Copenhagen, Denmark; 17 The Charles Bronfman Institute for Personalized Medicine, Icahn School of Medicine at Mount Sinai, New York, NY USA; 18 National Institutes of Health, Bethesda, MD USA; 19 Nanjing Medical University, Nanjing, China

**Keywords:** Clinical genetics, Risk factors

## Abstract

Polygenic risk scores (PRSs) have improved in predictive performance, but several challenges remain to be addressed before PRSs can be implemented in the clinic, including reduced predictive performance of PRSs in diverse populations, and the interpretation and communication of genetic results to both providers and patients. To address these challenges, the National Human Genome Research Institute-funded Electronic Medical Records and Genomics (eMERGE) Network has developed a framework and pipeline for return of a PRS-based genome-informed risk assessment to 25,000 diverse adults and children as part of a clinical study. From an initial list of 23 conditions, ten were selected for implementation based on PRS performance, medical actionability and potential clinical utility, including cardiometabolic diseases and cancer. Standardized metrics were considered in the selection process, with additional consideration given to strength of evidence in African and Hispanic populations. We then developed a pipeline for clinical PRS implementation (score transfer to a clinical laboratory, validation and verification of score performance), and used genetic ancestry to calibrate PRS mean and variance, utilizing genetically diverse data from 13,475 participants of the All of Us Research Program cohort to train and test model parameters. Finally, we created a framework for regulatory compliance and developed a PRS clinical report for return to providers and for inclusion in an additional genome-informed risk assessment. The initial experience from eMERGE can inform the approach needed to implement PRS-based testing in diverse clinical settings.

## Abstract (web summary)

A new study from the eMERGE consortium provides insights on the development of a pipeline for the generation and reporting of polygenic risk scores for ten diseases to diverse populations in a clinical setting.

## Main

Polygenic risk scores (PRSs) aggregate the effects of many genetic risk variants and can be used to predict an individual’s genetic predisposition to a disease or phenotype^1^. PRSs are being calculated and disseminated at a prodigious rate^1,2^, but their development and application to clinical care, particularly among ancestrally diverse individuals, present substantial challenges^3–5^. Incorporation of genomic risk information has the potential to improve risk estimation and management^4,6^, particularly at younger ages^7^. Clinical use of PRSs may ultimately prevent disease or enable its detection at earlier, more treatable stages^7–10^. Improved estimation of risk may also enable targeting of preventive or therapeutic interventions to those most likely to benefit from them while avoiding unnecessary testing or overtreatment^10,11^. However, clinical use of Eurocentric PRSs in diverse patient samples risks exacerbating existing health disparities^12–14^.

PRSs for individual conditions are typically generated from summary statistics derived from genome-wide association studies (GWASs), which are themselves derived from populations that are heavily overrepresented by individuals of European ancestry^12^. Such scores have been shown to have limited prediction accuracy with increasing genetic distance from European populations^12,15^. PRSs can be improved if developed and validated using multiancestry cohorts^16^. Clinical and environmental data combined with monogenic and polygenic risk measurements can improve risk prediction, as demonstrated in ref. ^17^ and other studies^18^. Approaches for combining genomic and nongenomic information, optimizing models for populations of diverse genetic ancestry and across age groups, and conveying this information to clinicians and patients have yet to be developed and applied in clinical care. Various forms of PRSs are available to consumers through commercial platforms such as 23andMe, Myriad Genetics (riskScore), Allelica, Ambry Genetics, and others, and several noncommercial studies have explored the clinical use of PRSs in direct-to-participant models^19–21^; however, there is limited information on the clinical implementation considerations of returning PRSs across multiple phenotypes in a primary care setting^20^. Even before assessing the ability of PRSs to improve health outcomes, reduce risk and enhance clinical care, large multicenter prospective pragmatic studies are needed to assess how patients and care providers interact with and respond to PRSs in a primary care setting^22^.

The Electronic Medical Records and Genomics (eMERGE) Network is a multicenter consortium established in 2007 to conduct genomic research in biobanks with electronic medical records^23,24^. In 2020, eMERGE embarked on a study of genomic risk assessment and management in 5,000 children and 20,000 adults of diverse ancestry, beginning with efforts to identify and validate published PRSs across multiple race-ethnic groups (and inferred genetic ancestries) in ten common diseases with complex genetic etiologies. The study plans for 25,000 individuals (aged 3–75 years) to be recruited from general healthcare system populations. Six of the ten recruitment sites are committed to recruiting an ‘enhanced diversity cohort’, meaning that their enrollment will target 75% of enrolled individuals belonging to a racial or ethnic minority or medically underserved population, whereas the remainder of clinical sites will target 35% minority participants^22^. Enrollment is not targeted to individuals with specific conditions, although individuals with prevalent conditions can be included. For this prospective, pragmatic study, the primary outcome being measured is the number of new healthcare actions after return of the genome-informed risk assessment. This paper describes (1) identification, selection and optimization of the PRSs that are included in the study; (2) calibration of ancestry for PRS estimation using a modified method developed for eMERGE; (3) development and launch of clinical reporting tools; and (4) an overview of the first 2,500 samples processed as part of the study.

## Results

### PRS auditing and evaluation

**Fig. 1:**
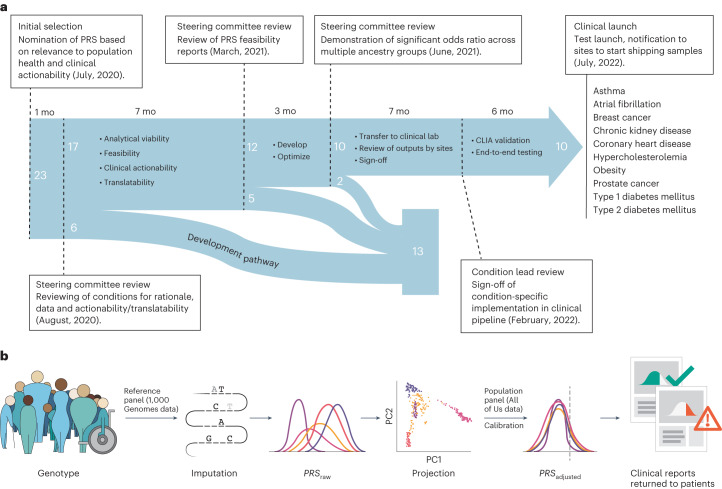
Timeline and process overview. **a**,Timeline and process for selection, evaluation, optimization, transfer, validation and implementation of the clinical PRS test pipeline. Dashed lines represent pivotal moments in the progression of the project with duration between these events indicated in months (mo) above the blue arrow. Numbers in white represent the number of conditions being examined at each stage and their fates. List of ten conditions on the right-hand side indicates the conditions that were implemented in the clinical pipeline for this study. **b**, Overview of the eMERGE PRS process. Participant DNA is genotyped using the Illumina Global Diversity Array, which assesses 1.8 million sites. Genotyping data are phased and imputed with a reference panel derived from the 1,000 Genomes Project. For each participant, raw PRSs are calculated for each condition (*PRS*_raw_). Each participant’s genetic ancestry is algorithmically determined in the projection step. For each condition, an ancestry calibration model is applied to each participant’s *z-*scores based on model parameters derived from the All of Us Research Program (Calibration) and an adjusted *z*-score is calculated (*PRS*_adjusted_). Participants whose adjusted scores cross the predefined threshold for high PRS are identified and a pdf report is generated. The report is electronically signed after data review by a clinical laboratory director and delivered to the study portal for return to the clinical sites.

To select the PRSs for clinical implementation, the Network conducted a multistage process to evaluate proposed scores (Fig. 1). An initial set of 23 conditions was selected based on considerations including relevance to population health (condition prevalence and heritability), strength of evidence for PRS performance, clinical expertise in the eMERGE Network, and data availability that would facilitate validation of the PRS in diverse populations. These conditions were abdominal aortic aneurysm, age-related macular degeneration, asthma, atopic dermatitis, atrial fibrillation, bone mineral density, breast cancer, Crohn’s disease, chronic kidney disease, colorectal cancer, coronary heart disease, depression, hypercholesterolemia, hypertension, ischemic stroke, lupus, nonalcoholic fatty liver disease, obesity, primary open angle glaucoma, prostate cancer, rheumatoid arthritis, type 1 diabetes and type 2 diabetes.

Network sites completed a comprehensive literature review on 23 proposed conditions and the corresponding PRSs. A summary of the features of the PRS for each of the final conditions chosen is shown in Supplementary Table 1. The collated information included analytic viability—a description of covariates, the age, and ancestry effects of the original PRS model; feasibility—access to sufficiently diverse validation datasets (genetic ancestry and age) as well as condition prevalence and relevance to preventative care; potential clinical actionability—existing screening or treatment strategies, and magnitude (odds ratio) of risk in the high-risk group; and translatability—expected public health impact across diverse populations. Candidate PRSs were restricted to those that were either previously validated and published (journal or preprint) or for which there was sufficient access to information to develop and/or optimize new PRSs, which could then be validated.

In auditing and evaluating evidence of PRS performance, the eMERGE steering committee considered PRSs for conditions that could be implemented in pediatric and/or adult populations, and for diseases with a range of age of onset (0 to >65 years of age). We considered published single nucleotide polymorphism (SNP)-based heritability estimates available for ten of the 23 conditions, ranging from 3% to 58%. The majority of PRSs under consideration aimed to identify individuals at high risk for disease; however, PRSs to predict disease severity and drug response were also considered. Two of the conditions, breast cancer and prostate cancer, were only considered for implementation in individuals whose biological sex was female or male, respectively. As the eMERGE Network plans to enroll >50% participants from underrepresented groups (including racial and ethnic minority groups; people with lower socioeconomic status; underserved rural communities; sexual and gender minority groups)^25^, emphasis was placed on PRSs that were already available for, or could be developed and validated in, diverse population groups.

To define population groups, study-level population descriptors were first extracted from published literature, preprints or information shared directly by collaborators on data used to develop and/or optimize and/or validate PRSs. Methods for defining population groups across studies ranged from self-reporting, extraction from health system data and/or analysis of genetic ancestry. We designated four population groups: European ancestry (that is, study population descriptors included European, European-American or other European descent diaspora groups), African (African, African American or other African descent diaspora groups), Hispanic (that is, Hispanic, Latina/o/x or those who have origins in countries in the Caribbean and Latin America) and Asian (that is, South Asian, East Asian, South-East Asian, Asian-American or other diaspora Asian groups).

Thirteen conditions were considered and not selected for clinical implementation (Fig. 1). Of the six conditions dropped from consideration in August 2020, low disease prevalence across ancestral groups (age-related macular degeneration), availability of diverse genetic datasets for validation (primary open angle glaucoma, rheumatoid arthritis and Crohn’s disease) and the lack of a validated algorithm to identify patients and controls based upon electronic health record (EHR) (bone mineral density) were the driving factors. In March 2021, five additional conditions were dropped from consideration for clinical implementation based upon the progress of the development and validation of a multiancestral PRSs (depression, ischemic stroke), the low predictive value of candidate PRSs (hypertension, nonalcoholic fatty liver disease) and ethical considerations around returning results to a condition with low population prevalence (lupus).

Conditions not prioritized for implementation continued on a ‘developmental’ pathway for further refinement. Each of the 12 conditions that were selected to move forward from the March 2021 review was assigned a ‘lead’ and ‘co-lead’ site, which worked together to develop, validate and transfer the score to the clinical laboratory for instantiation and Clinical Laboratory Improvement Amendments (CLIA) validation. Assignment of leads was based on site preference, expertise and distribution of workload.

### Selection, optimization and validation

A systematic framework was developed to evaluate the performance for the remaining 12 PRSs, in accordance with best practices outlined in ref. ^26^. An in-depth evaluation matrix of the 12 chosen conditions can be found in Supplementary Table 2. The Network carefully considered a variety of strategies to optimize PRS generalizability and portability. The Network prioritized validation across four ancestries with an emphasis on African and Hispanic ancestry due to their underrepresentation in genetic research and projected representation within the study cohort. We determined that a PRS was validated if the odds ratios were statistically significant in a minimum of two and up to four ancestral populations: African/African American, Asian, European ancestry, and Hispanic/Latino. The PRS Working Group members conducted an extensive scoping exercise to identify suitable datasets of multiple ancestries for disease-specific PRS validation. These included datasets from early phases of eMERGE (2007–2019) as well as external datasets such as the UK Biobank and Million Veteran Program. These larger population-level databases had the advantage of large sample sizes and less case–control ascertainment bias (though other sources of bias can still be an issue; ‘Discussion’). A standardized set of questions was addressed by the disease leads that included the source of discovery and validation datasets, the availability of multiancestry validation datasets, the availability of cross-ancestry PRSs (that is, PRS models that were developed and validated in more than one genetic ancestry), proposed percentile thresholds for identifying high-risk status, model discrimination (AUC) and effect sizes (odds ratios) associated with high-risk versus not high-risk status (Supplementary Table 2). For seven out of the 12 candidate scores, no further optimization of the original model was performed. For five scores, an additional optimization effort was undertaken to further refine the score performance in multiple ancestries. Details of the optimization can be found in Supplementary Table 3. A specific score optimization was applied for chronic kidney disease. This optimization consisted of adding the effect of *APOL1* risk genotypes to a polygenic component, which has been found to improve risk predictions in African ancestry cohorts^27^.

For the final selection of PRSs to be included in the prospective clinical study, the steering committee considered the score performance summaries (presented by condition leads) in addition to the actionable and measurable recommendations relevant for return, for each condition, in the prospective cohort. Abdominal aortic aneurysm was removed from the clinical pathway in June 2021 based on inability to pull a critical risk factor from the EHR (smoking) and a relatively low disease prevalence in Asian and Hispanic populations. Colorectal cancer was removed in June 2021 because the development and validation of the PRS was not complete for all the ancestral groups (Fig. 1). For the ten remaining phenotypes, the prospective pragmatic study required a small number of measurable primary clinical recommendations per phenotype so that the utility of the PRS to change physician and patient behavior can be measured. These recommendations can be found in Supplementary Tables 2 and 4 of ref. ^22^.

### Population-based *z*-score calibration

In this study, the focus is on integration and implementation of validated PRSs in clinical practice rather than novel PRS development. Ultimately, the Network opted to balance generalizability and feasibility by validating and returning cross-ancestry PRSs. However, even with cross-ancestry scores, differences remain in the distribution of *z*-scores for the PRSs across genetic ancestries that can result in inconsistent categorization of individuals into ‘high’ or ‘not high’ polygenic risk categories for a given condition^28^. To that end, the Network chose to develop methods to genetically infer each participant’s ancestry and calibrate the distribution of resulting *z*-scores through a population-based calibration model^28,29^ (see below). An alternative would have been to apply existing PRSs in available samples of different ancestries and derive ancestry-specific effect estimates. However, returning ancestry-specific risk estimates is challenging in real-world implementations as it would require self-reporting of ancestry by patients (who may not be able to provide this with accuracy) and developing multiple ancestry-specific reports for each health condition. In addition, such PRSs would be problematic to return to patients of mixed ancestry.

PRSs often have different means and standard deviations for individuals from different genetic ancestries. While some of these differences could be due to true biological differences in risk, they also result from allele frequency and linkage disequilibrium structure differences between populations^30^. This problem is more acute when a PRS is calculated for an individual whose ancestry does not match the ancestries used to develop the PRS. A clinically implemented PRS test to return disease risk estimates, therefore, must be adjusted to account for these differences due to ancestral background. A calibration method based on principal component analysis (PCA), which was initially described in ref. ^28^, was modified to model both the variance and means of scores as ancestry dependent, as compared to the previous method (Methods), which modeled only the means as dependent on ancestry. This modification was found to be necessary because some conditions were found to exhibit highly ancestry-dependent variance, which would have led to many more or fewer participants of certain ancestries receiving a ‘high PRS’ determination than was intended. One option considered to create and train the calibration model was to enroll and process a representative number of participants then pause on the return of results while the model was trained and the older data reprocessed. This stop–start approach was deemed suboptimal. Instead, the model was fit, with permission, to a portion of the All of Us (AoU) Research Program (https://www.researchallofus.org/) cohort genotyping data, which allowed for continuous return of results to eMERGE participants once the study began. Of note, the All of Us Research Program cohorts selected for both training and testing the calibration model exhibited high degrees of genetic admixture, which would be expected to accurately reflect the study enrollment population. Importantly, because no ancestry group is homogenous, when individuals are compared directly to other individuals in their assigned population group, a dependence between admixture fraction and PRS can result. This dependence is removed by the described PCA calibration method, and the resulting calibrated PRSs are independent of admixture fraction. More details about the ancestry calibration can be found in Methods.

### Transfer and implementation

**Fig. 2:**
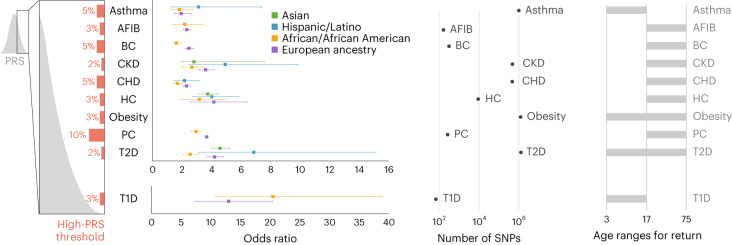
Summary of the ten conditions that were implemented. ‘High-PRS threshold’ represents the percentile that is deemed to be the cutoff for a specific condition above which a high-PRS result is reported for that condition. Odds ratios are reported as the mean odds ratios (square dot) associated with having a score above the specified threshold, compared to having a score below the specified threshold, along with 95% confidence intervals (CIs), shown in the whiskers. The number of case and control samples used to derive these odds ratios and CIs for each condition can be found in Supplementary Table 2. Note that the odds ratio for obesity is not reported here, as it will be published by the Genetic Investigation of ANthropometric Traits consortium (Smit et al., manuscript in preparation). ‘Number of SNPs’ represents the range of numbers or sites included in each score. ‘Age ranges for return’ indicates the participant ages at which a PRS is calculated for a given condition. AFIB, atrial fibrillation; BC, breast cancer; CKD, chronic kidney disease; CHD, coronary heart disease; HC, hypercholesterolemia; PC, prostate cancer; T1D, type 1 diabetes; T2D, type 2 diabetes.

Once the final ten conditions had been selected, condition leads worked with computational scientists at the clinical laboratory (Clinical Research Sequencing Platform, LLC at the Broad Institute) to transfer the PRS models and create the sample and data-processing workflow (Fig. 2). Condition-specific models were run with outputs from the lab’s genotyping (Illumina Global Diversity Array (GDA)), phasing (Eagle2 (ref. ^31^) https://github.com/poruloh/Eagle) and imputation (Minimac4 (ref. ^32^) https://genome.sph.umich.edu/wiki/Minimac4) pipelines to assess genomic site representation (see Methods for more information on the architecture and components of the pipeline). Several rounds of iteration between the clinical laboratory and condition leads followed in which any issues with the pipeline were resolved and the effect of genomic site missingness was assessed (Table 1). The final version of the implemented models was returned to the condition leads to recalculate effect sizes in the validation cohorts.

**Table 1:** Performance measures from the PRS pipeline validation study at the clinical laboratory

		Asthma	Atrial fibrillation	Breast cancer	Chronic kidney disease	Coronoary heart disease	Hypercholesterolemia	Obesity/BMI	Prostate cancer	Type 1 diabetes	Type 2 diabetes
PRS accuracy: Pearson correlation between PRS from array and WGS (%)		99.3	98.6	93.0	98.3	98.2	95.9	99.5	96.4	99.5	98.8
PRS precision: PRS pipeline repeatability (%)		100	100	100	100	100	100	100	100	100	100
PRS precision: PRS pipeline reproducibility (*z*-score standard deviation)		0.0020	0.0010	0.0040	0.0001	0.0010	0.0050	0.0020	0.0006	0.0001	0.0010
PRS site missingness (%)		0.69	1.20	0.32	0.69	0.46	1.20	0.70	2.97	2.97	0.70
Odds ratio (95% CI)	European	1.95 (1.43–2.65)	2.32 (2.07–2.61)	2.47 (2.20–2.77)	3.6 (3.11–4.17)	2.3 (2.07–2.56)	4.16 (2.59–6.44)		3.67 (3.57–3.76)	12.97 (7.29–20.40)	4.21 (3.66–4.84)
	African American	1.83 (1.24–2.70)	2.19 (1.38–3.38)	1.61 (1.38–1.87)	2.66 (2.01–3.51)	1.68 (1.39–2.03)	3.16 (1.92–5.01)		2.95 (2.60–3.30)	20.45 (10.77–38.83)	2.55 (2.09–3.11)
	Hispanic	3.12 (1.32–7.44)	2.27 (1.09–4.50)	2.05 (1.10–3.83)	4.93 (2.46–9.89)	2.16 (1.47–3.19)	4.02 (2.72–5.83)		n.d.	n.d.	6.87 (3.11–15.15)
	Asian	n.d.	n.d.	2.22 (1.99–2.47)	3.81 (1.91–7.59)	n.d.	3.75 (3.15–4.42)		n.d.	n.d.	4.58 (4.00–5.23)

PRS pipeline accuracy is assessed as the Pearson correlation between scores derived from polymerase chain reaction (PCR)-free 30X whole genome sequencing (WGS) and those derived from imputed genotyping data (GDA) in the same 70 specimens. Pearson correlation is shown in the mean correlation across all ancestry groups tested. PRS pipeline precision (repeatability) is the measure of concordance in PRSs calculated from the same 70 specimens, run through the pipeline ten times over the course of two weeks. PRS pipeline precision (reproducibility) is assessed using three samples, each run six times end-to-end and then compared in a pairwise manner. The *z*-score standard deviation is used as a measure of variability. PRS site missingness is the percentage of genomics sites in the original score that are missing from the final imputed dataset. Odds ratios for high PRS versus not high PRS are derived from the condition-specific cohorts and calculated by each condition group lead across the ancestries available. Odds ratio information for obesity/BMI is in preparation for publication by the Genetic Investigation of ANthropometric Traits consortium. BMI, body mass index; CI, confidence interval; n.d., no data.

Finally, as part of the implementation of the PRS pipelines as a clinical test in a CLIA laboratory, a validation study was performed (see Methods for a detailed description; Table 1 summarizes some of the results). Briefly, this study leveraged 70 reference cell lines from diverse ancestry groups (Coriell) where 30X whole genome sequencing data were generated to form a variant truth set from which the technical accuracy and reproducibility of imputation and PRS calling was assessed. A second sample set of 20 matched donor blood and saliva specimens was procured to assess the performance of the pipeline with different input materials. A set of three samples, each with six replicates, was run end-to-end through the wet lab and analytical pipelines as an assessment of reproducibility. As a verification of the clinical validity of the scores, cohorts of cases for eight of the ten conditions were created using the eMERGE phase III imputed dataset (available on https://anvil.terra.bio/#workspaces/anvil-datastorage/AnVIL_eMERGE_GWAS/data (registration required)). PRS performance measures were calculated to confirm associations between scores and conditions. Due to limitations in the eMERGE phase III imputation (no chromosome X, different imputation pipeline), the odds ratios from this analysis were not included in the final reports; rather, the odds ratios calculated in the condition-specific validation cohorts (using the final clinical lab pipeline) were used (Fig. 2 and Table 1). A validation report was created for each condition. This report was reviewed and approved by the Laboratory Director in compliance with CLIA regulations for the development of a laboratory-developed test. Personnel were trained on laboratory and analytical procedures, and standard operating procedures were implemented. Data review metrics were established, sample pass/fail criteria were defined, and order and report data-transfer pipelines were built as described in ref. ^22^.

### Creation of pipeline for report creation, review, sign-out and release

A software pipeline was built to facilitate the data review and clinical report generation. Reports were created both as documents (in pdf format) and structured data (in JSON format; a sample report is included in the Supplementary Information). Automated rules for case triage were built into the PRS calculation and reporting pipeline to account for differences in return based on age and sex at birth for certain conditions. For instance, the PRS for breast cancer is only calculated for participants who report sex at birth as female; similarly, prostate cancer scores are only generated for participants who report sex at birth as male. Age-related restrictions were similarly coded into the pipeline to account for study policies on return. Data review by an appropriately qualified, trained individual is required for high complexity clinical testing. In the PRS clinical pipeline, this review takes the form of a set of metrics that are exposed by the pipeline to the reviewer. These include a *z*-score range for each condition (passing samples will have a score −5 < *z* < +5), a PCA plot per batch against a reference sample set (visual representation of outlier samples), monitoring the *z*-score range for each control per condition (one control on each plate; NA12878) and flagging any samples with multiple ‘high risk’ results for further review.

Each participant’s sample is also run on an orthogonal fingerprinting assay (Fluidigm biomark) that creates a genotype-based fingerprint for that DNA aliquot. Infinium genotyping data are compared to this fingerprint as a primary check of sample chain-of-custody fidelity and to preclude sample or plate swaps during lab processing. Reviewed and approved data for a participant are processed into a clinical report. The text and format of this report were created during an iterative review process by consortium work groups. For this pragmatic clinical implementation study, two results are returned to participants: ‘high risk’ or ‘not high risk’ based on the PRS^22^. In the clinical report, a qualitative framework has been developed to indicate for which condition(s) a participant has been determined to have a high PRS (if any). Quantitative values (*z-*scores) are not included for any condition in the main results panel. For breast cancer and CHD, the *z*-score is presented in another section of the report for inclusion in integrated score models for those conditions. For breast cancer specifically, the provided *z*-score is used with the BOADICEA^33^ model to generate an integrated risk that is included in the genome-informed risk assessment (GIRA), as described in ref. ^22^.

### Overview of the first 2,500 clinical samples processed

**Fig. 3:**
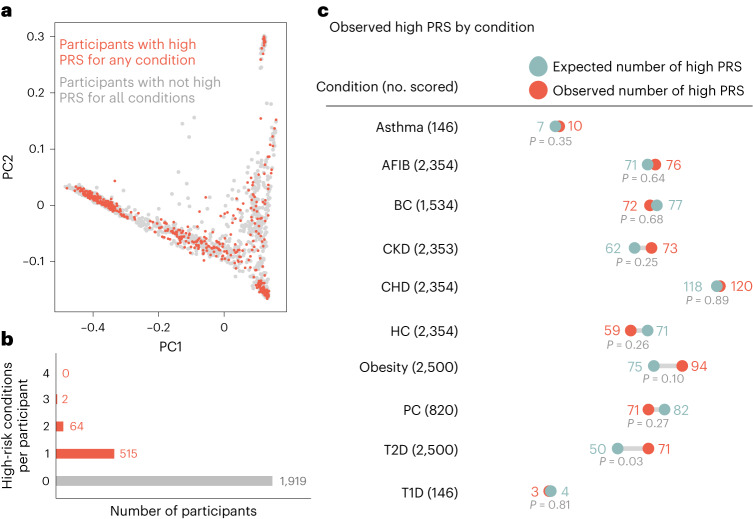
Summary of the first 2,500 eMERGE participants processed through the clinical pipeline. **a**, PCA of ancestry indicating participants with a result of ‘high PRS’ for any condition (red dots) compared to participants who did not have a high PRS identified (gray dots). **b**, Summary of number of high-risk conditions found per participant. **c**, Observed numbers of high PRS called per condition compared to the expected numbers of high PRS per condition. *P* values are two-sided *P* values calculated by simulation to account for the uncertainty in the All of Us (AoU) derived ancestry calibration parameters due to the finite size of the AoU training cohort, and further adjusted for multiple hypothesis testing using the Holm–Šidák procedure. Note not all participants get scored for every condition based on age and sex at birth filters.

Between the launch in July 2022 and May 2023, 2,500 participants were processed through the clinical PRS pipeline (representing ∼10% of the proposed cohort). Of the first 2,500 participants processed, 64.5% (1,612) indicated sex at birth as female, while 35.5% (886) indicated male. Median age at sample collection was 51 years (range: 3 years to 75 years). Participants self-reported race/ancestry, with 32.8% (820) identifying as ‘White (for example, English, European, French, German, Irish, Italian, Polish, etc.)’; 32.8% (820) identified as ‘Black, African American or African (for example, African American, Ethiopian, Haitian, Jamaican, Nigerian, Somali, etc.)’; 25.4% (636) identified as ‘Hispanic, Latino or Spanish (for example, Colombian, Cuban, Dominican, Mexican or Mexican American, Puerto Rican, Salvadoran, etc.)’; 5% (124) identified as ‘Asian (for example, Asian, Indian, Chinese, Filipino, Japanese, Korean, Vietnamese, etc.)’; 1.5% (38) identified as American Indian or Alaska Native (for example, Aztec, Blackfeet Tribe, Mayan, Navajo Nation, Native Village of Barrow (Utqiagvik) Inupiat Traditional Government, Nome Eskimo Community, etc.); 0.9% (22) identified as Middle Eastern or North African (for example, Algerian, Egyptian, Iranian, Lebanese, Moroccan, Syrian, etc.); 0.8% (21) selected ‘None of these fully describe [me_or_my_child]’; 0.7% (17) selected ‘Prefer not to answer’; 0.1% (2) participants had incomplete data. A summary of the performance of the first 2,500 samples and resulting high-PRS metrics are shown in Fig. 3. In the first 2,500 participants, we identified 515 participants (20.6%) with a high PRS for one of the ten conditions, 64 participants (2.6%) had a high PRS for two conditions, and two participants (0.08%) had a high PRS for three conditions. The remaining 1,919 participants had no high PRS found. High-PRS participants spanned the spectrum of genetic ancestry when projected onto principal component space (Fig. 3). Observed numbers of high-PRS assessments were largely consistent with the corresponding expected numbers. The *P* values in Fig. 3c are two-sided *P* values, which are calculated taking into account both the finite size of the eMERGE cohort and the finite size of the training data used to estimate the ancestry adjustment parameters. The *P* values are further adjusted for multiple hypothesis testing using the Holm–Šidák procedure^34^.

## Discussion

While the predictive performance of PRSs has improved substantially in recent years, challenges remain in ensuring that PRSs are applicable and effective in diverse populations. In particular, the vast majority of GWASs have focused on individuals of European ancestry, and the predictive accuracy of PRSs declines with increasing genetic distance from the discovery population^5,30,35^. This risks exacerbating existing health disparities, as clinical use of Eurocentric PRSs in diverse patient samples may not accurately reflect disease risk in non-European populations. To address these challenges, the eMERGE Network has conducted a multistage process to evaluate and optimize PRS selection, development and validation. The Network has prioritized conditions with high prevalence and heritability, existing literature, clinical actionability and the potential for health disparities, and has developed strategies to optimize PRS generalizability and portability across diverse populations. In particular, the Network has emphasized performance across four major ancestry groups (African, Asian, European, Hispanic, as reflected by self-identified race/ethnicity) and has developed a pipeline for clinical PRS implementation, a framework for regulatory compliance and a PRS clinical report.

The potential impact of PRS-based risk assessment in clinical practice is substantial. By enabling targeted interventions and preventative measures, PRS-based risk assessment has the potential to reduce the burden of a range of conditions^22^. Moreover, the development of PRS-based risk assessment in diverse populations has the potential to reduce health disparities by ensuring that clinical use of PRSs accurately reflects disease risk in diverse populations.

However, challenges remain in the successful implementation of PRS-based risk assessment in clinical practice. Participation bias in training or validation datasets that do not accurately represent the broader populations, for example the United Kingdom BioBank, can lead to skewed results and reduced generalizability in PRS test development^36^. Other challenges include concerns about genetic determinism, the potential for stigmatization and the need for robust regulatory frameworks to ensure that PRS-based risk assessment is deployed safely and effectively. Furthermore, to have more clinical utility, an individual’s PRS-based risk would be calculated as age-based absolute risk. Challenges also remain in healthcare provider and patient understanding and interpretation of PRS results and how to effectively communicate these results. Additionally, one of the biggest challenges is the implementation of effective disease prevention strategies after the return of the results. Return of the results will not result in a benefit without effective disease prevention or early detection strategies. The eMERGE Network’s work provides a blueprint for addressing these challenges, but ongoing research and evaluation will be necessary to ensure that PRS-based risk assessment is implemented in a responsible and effective manner. While this study will not answer all of the unanswered challenges and questions, the results from the 25,000 subjects from the eMERGE study will provide additional data to existing risk stratification to model harms and benefits over patient lifetimes.

Future groups developing, transferring and implementing PRSs into a clinical setting could build upon the eMERGE experience. Slightly less than half of the phenotypes originally considered for PRS development were able to be continued through clinical implementation based on varying considerations, suggesting that a moderately high number of phenotypes with measurable genetic contributions will be appropriate for PRS-based clinical tools. Thresholds for returning ‘high risk’ PRS were identified by each phenotype working group based in part upon the statistical significance between the ‘high-risk’ and ‘not high-risk’ groups. Future studies might consider standardizing the analyses and methods used to define these thresholds. Additionally, to have more clinical utility, an individual’s PRS-based risk would be calculated as an age-based absolute risk. While data for these risk assessments are available for some phenotypes (for example, cardiovascular and cancer), age of onset data are lacking for many clinically important phenotypes. Finally, the standards, guidance and the development of best practices for the integration of PRSs into clinical processes are yet to be developed. Future studies can learn from eMERGE and other groups' experiences will be a foundation for ongoing opportunities for the integration of polygenic risk predictions in clinical care settings.

In conclusion, the eMERGE Network’s work in PRS development represents an important step forward in the implementation of PRS-based risk assessment (in combination with other risk estimates from monogenic testing and family history) in clinical practice.

## Methods

### Consent and ethical approval

The study was conducted in accordance with the Declaration of Helsinki, and the central institutional regulatory board protocol was approved by the Ethics Committee of Vanderbilt University. All participants for eMERGE are consented, using a global primary consent and a site-specific consent. Minors acknowledge study participation by signing an assent (if local policy dictates) and the child’s parent/guardian signs a parental permission form. The Vanderbilt University Medical Center Co-ordinating Center is the institutional review board of record (no. 211043) for the Network’s single institutional review board, approved in July 2021.

For the All of Us Research Program, informed consent for all participants is conducted in person or through an eConsent platform that includes primary consent, Health Insurance Portability and Accountability Act authorization for research EHRs and consent for return of genomic results. The protocol was reviewed by the Institutional Review Board (IRB) of the All of Us Research Program. The All of Us Institutional Review Board follows the regulations and guidance of the National Institutes of Health Office for Human Research Protections for all studies, ensuring that the rights and welfare of research participants are overseen and protected uniformly.

### Clinical trials registration

The eMERGE genomic risk assessment study is a registered, prospective, interventional clinical trial registered with clinicaltrials.gov (Identifier: NCT05277116). The purpose of the study is to determine if providing a GIRA will impact clinical actions taken by providers and patients to manage disease risk and the propensity of participants to develop a disease reported in the GIRA. For this prospective, pragmatic study, the primary outcome being measured is the number of new healthcare actions after return of the genome-informed risk assessment. Number of new healthcare actions will be measured by electronic health record data and participant-reported outcomes through a REDCap survey. Prespecified actions will include a condition-specific composite of new encounters, clinical orders or specialty referrals for clinical evaluation associated with the condition(s), placed by a provider within six months of result disclosure.

Secondary outcomes are the number of newly diagnosed conditions after return of the genome-informed risk assessment and the number of risk-reducing interventions after return of the genome-informed risk assessment (time frame: six months and 12 months post return of results to participant).

### Population group definition

In the score auditing and evaluation phase, condition leads cataloged population groups used in the development or validation of given scores from available publications, preprints or information shared directly from collaborators. Across the initial list of evaluated scores, methods for defining population groups included self-reporting, extraction from health system data and/or analysis of genetic ancestry. In the optimization phase, populations were defined using either computational analysis alone or both computational analysis and self-reported ancestry, as indicated in Supplementary Table 3. For creation of the training model for PRS ancestry calibration, populations were computationally determined as described in ‘PRS ancestry calibration overview’ below.

Populations with that are underserved and more frequently experience health disparities include racial and ethnic minority groups; people with lower socioeconomic status; underserved rural communities; sexual and gender minority groups; and people with disabilities^25^.

### Analytical and technical validation studies

#### Broad imputation pipeline overview

An imputation pipeline that takes as an input a variant call format (VCF) file generated from a genotyping microarray and imputes the genotypes at additional sites across the genome was developed. The pipeline architecture and composition was based on the widely used University of Michigan Imputation Server, which uses a software called Eagle (https://github.com/poruloh/Eagle) for phasing and Minimac4 (https://genome.sph.umich.edu/wiki/Minimac4) for the imputation. The pipeline uses a curated version of the 1,000 Genomes Project (1KG, www.internationalgenome.org) as the reference panel. Additional details on the imputation pipeline can be found at https://broadinstitute.github.io/warp/docs/Pipelines/Imputation_Pipeline/README.

#### Broad curated 1KG reference panel

During the validation process, we determined that some sites in the 1KG reference panel were incorrectly genotyped compared to the sites in matching whole genome sequencing data. To increase accuracy of the imputation and PRS scoring, we curated the original panel by removing sites that were likely incorrectly genotyped based on comparing allele frequencies to those reported in gnomAD v.2 (https://gnomad.broadinstitute.org/). Documentation of this curation can be found at https://broadinstitute.github.io/warp/docs/Pipelines/Imputation_Pipeline/references_overview and a publicly available version of the panel at the following Google Cloud location (accessible via the gsutil utility): gs://broad-gotc-test-storage/imputation/1000G_reference_panel/.

Selection of a reference panel for imputation as an input to a PRS is an important consideration. Some reference panels (for example, Trans-Omics for Precision Medicine (TOPMed)) have more samples than the default used in our pipeline (that is, 1KG). This leads to more variants being imputed. The question is whether this would materially change the PRSs calculated from samples imputed with the TOPMed panel. Access to this panel computationally is restricted (and local download prohibited) so it was deemed infeasible to implement in our clinical production environment. The performance of a non-eMERGE PRS (for CHD; ref. ^28^) using the two different reference panels was determined for 20 GDA saliva specimens and for 42 AoU array v.1 specimens. The cohort was imputed both by the Broad imputation pipeline with curated 1KG as the reference panel as well as on the TOPMed imputation server with TOPMed as the reference panel. Imputed arrays were scored by the PRS pipeline.

The PRS percentiles computed with each method are highly concordant for both cohorts. The Pearson correlation coefficient is 0.996 for both cohorts, the *P* value of the Welch two-sample *t-*test is equal to 0.93 and 0.85 (indicating no statistical difference between the methods) for GDA and AoU v.1 cohorts, respectively.

#### Performance verification of the imputation pipeline

Imputation accuracy was determined for 42 specimens that were processed through a genotyping microarray (AoU v.1 array—the precursor to the commercial Global Diversity Array) and imputed with curated 1KG as the reference panel where corresponding deep-coverage (>30X) PCR-free whole genome sequencing data were used as a truth call set to calculate sensitivity and specificity. The arrays were also imputed on the Michigan Imputation Server with 1KG as the reference panel.

Within the cohort, four different ancestries were represented: non-Finnish Europeans, East Asians, South Asian (SAS), African (AFR). Broad imputation pipeline sensitivity for SNPs is >97% and insertions/deletions (INDELs) >95% for all ancestries. Similarly, specificity for SNPs from the Broad imputation pipeline is above 99% and the specificity for INDELs is >98%. See Extended Data Table 1 for a table of results. Results were highly concordant with those returned by the remote server at Michigan.

#### Performance evaluation of different input material types

To assess the performance of specimens derived from both saliva and whole blood, a set of 20 matched blood and saliva pairs were run through the GDA genotyping process and the resulting VCFs were imputed using the Broad pipeline to be compared against results for matched blood-derived whole genome data. The Pearson correlation between sensitivity and specificity of blood- and saliva-derived samples are equal to 100% and 100%, respectively. For the same pairs, the Welch two-sample *t-*test statistic is 0.997 and 0.987, respectively. There is no significant difference between the different input sample types.

#### Imputation repeatability and reproducibility

Imputation pipeline repeatability was assessed by repeating imputation of a cohort of 1,000 Global Screening Array arrays ten times over the course of two weeks and was found to be 100% concordant. Imputation pipeline precision (reproducibility) was also tested on technical replicates. Three individual samples derived from saliva were each genotyped six times, followed by an imputation in a cohort of all saliva-derived samples. In each set of technical replicates, all pairs and variants in each pair were compared (making a total of 45 pairs for which genotypes were compared). Reproducibility is measured using Jaccard scores. ‘Reproducibility over variants’ was calculated only over sites where at least one of the two replicates in a pair calls a non hom-ref genotype and was found to be 99.91% (95% CI 99.89–99.93) for SNPs and 99.87% (95% CI 99.85–99.90) for INDELs. ‘Reproducibility over all sites’ was calculated over all genotyped sites, including sites genotyped as hom-ref in both replicates and was found to be 100% (95% CI 100–100) for both SNPs and INDELs.

#### Imputation performance as a function of variant frequency

Because we expect accuracy to be impacted by the frequency of a variant in the population (rare variants are less likely to be in the reference panel and therefore less accurately imputed), we further subdivided the performance assessment by allele frequencies on two cohorts: 42 AoU v.1 arrays and 20 blood–saliva pairs of GDA arrays. Accuracy of imputation of variants as a function of population allele frequency performed as expected, with rare variants in the population not being as accurately represented. Imputation is more accurate for variants that are more frequently observed in the population (≥0.1 allele frequency (AF)).

#### Impact of genotyping array call rate on imputation performance

The impact of call rate on the imputation was assessed by generating a downsampled series of 42 arrays, each with call rates of 90%, 95%, 97% and 98%. Pearson correlation values for SNPs and INDELs were calculated across bins of allele frequencies, assessed against gnomAD common variants (AF > 0.1), for the cohorts with downsampled call rates. Call rates below 95% were found to produce suboptimal results. At this rate the mean *R*^2^ dosage score for sites with AF ≥ 0.1 was found to be 0.98% (95% CI 0.98–0.98) for both SNPs and INDELs compared to 0.99% for call rates of 97% and 98%.

#### Impact of imputation batch size on performance

Batch size effect of the imputation pipeline was assessed by imputing and analyzing arrays in a cohort of size 1,000 (randomly chosen), ten cohorts of size 100 (nonoverlapping subsets of the 1,000 cohort) and ten cohorts of size ten (nonoverlapping subsets of one of the 100 cohorts). Pearson correlations of dosage scores were calculated across bins for allele frequencies (assessed against gnomAD) for smaller cohorts versus larger cohorts. The data show that imputation is highly correlated across batch sizes with batches down to as few as ten samples, producing acceptable performance. The mean *R*^2^ correlation of dosage scores for sites with allele frequency greater or equal to 0.1 is above 0.97 in all cases both for SNPs and INDELs and increases to 0.98 for the larger studied cohorts. Increasing batch sizes produces very slight improvements in imputation but these are not significant and the choice of imputation batch size (above or equal to ten samples) can be made on practical and operational grounds.

#### Broad PRS pipeline overview

The PRS pipeline begins by calculating a raw score using plink2 (https://www.cog-genomics.org/plink/2.0/). For each condition, effect alleles and weights are defined for a set of genomic sites stored in a weights file. At each site, the effect allele dosage observed in the imputed VCF is multiplied by the effect weight in the weights file. The raw score is the sum of these products over all the specified sites.

#### Validation of technical and analytical performance of the PRS pipeline

For each of the ten conditions chosen by the consortium for clinical return, a validation study was performed to assess the technical and analytical performance as well as to verify the association between score and disease risk. See Extended Data Table 2 for a summary of the validation measures.

##### PRS pipeline accuracy

Accuracy of the pipeline was determined by calculating the Pearson correlation between PRSs calculated from 70 specimens imputed from GDA array data and PRSs of corresponding deep-coverage PCR-free whole genome sequencing data (used as a truth call set).

##### Input material performance

Accuracy of PRS scoring when different sample types (blood or saliva) are used as inputs was determined by comparing the PRSs from matched blood and saliva pairs collected from 20 individuals.

##### PRS pipeline repeatability

PRS pipeline repeatability was assessed by running the pipeline on the same dataset of 70 imputed GDA arrays ten times over the course of two weeks (without call caching). Scores generated from the different processing runs were compared to determine if there are any differences observed for a given PRS when the pipeline is run at different times.

##### PRS pipeline reproducibility

PRS pipeline precision (reproducibility) was assessed using three samples each run six times end-to-end and then compared in a pairwise manner. The *z*-score standard deviation is used as a measure of variability.

##### PRS site representation

The SNP weight sites that are not called during genotyping or imputation were determined. These are sites not present in the intersection of an imputed GDA array and the reference panel. Ideally, all sites required for PRS calculation are present either as genotyped or imputed sites; however, in practice, a small number of sites are not present due to differences in the data used to create the score and the specific array and imputation reference panel used in this study.

##### Performance verification using eMERGE I–III cohort

A cohort of samples with known phenotypic information was used to verify the relationship between PRS as determined by our pipeline and disease risk. For conditions where cases and controls could be identified in the eMERGE I–III cohort, we determined performance using metrics outlined in the ClinGen working group recommendations^26^. Specifically, we determined the PRS distributions for cases and controls, we examined the impact of ancestry adjustment on the distributions and we examined the relationship between observed and predicted risk. An example of this analysis (for T2D is shown below).

The T2D weight file used for PRSs in this validation report comes from a GWAS by Ge et al.^29^ where they reported that individuals in the top 2% of the PRSs in the population have an increased risk of developing T2D.

**Extended Data Fig. 1:**
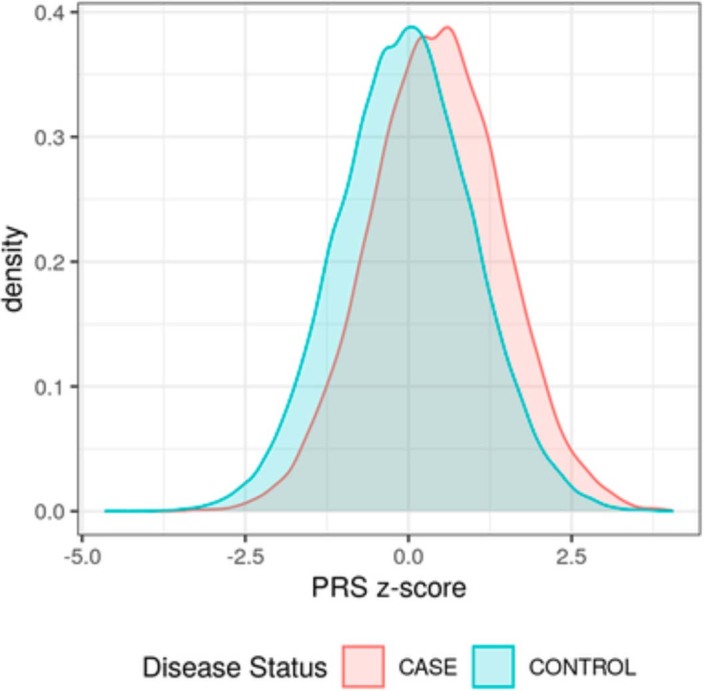
Case-control PRS histograms. Histograms of T2D PRS scores for case and control samples in the eMERGE I-III dataset.

The T2D cohort in the eMERGE I–III dataset consisted of 19,145 cases and 68,823 control samples. The mean adjusted PRS for case samples was 0.435, while the mean for control samples was −0.042. Individuals with higher adjusted PRS scores tend to be more likely to develop disease (see Extended Data Fig. 1 for a histogram of T2D PRSs in cases and controls).

There are some limitations to this analysis: (1) the eMERGE I–III dataset being used for this analysis was generated from different array platforms and was imputed with a different pipeline including a different version of the 1KG reference panel than the one currently implemented; (2) the eMERGE I–III imputed dataset does not include variants from chromosomes X or Y. For these reasons, the PRS disease association analysis represents a verification of the clinical validation performed by eMERGE condition leads rather than the quantitative measure of the impact of the score on risk. The clinical associations (odds ratios) that are reported on the clinical report for each condition were independently determined by eMERGE disease-specific expert teams.

##### Validation of pipeline and ancestry adjustment in original case–control cohorts

The final pipeline was made available to computational scientists at each of the eMERGE disease-specific expert teams who had access to appropriate case–control cohorts. These groups confirmed the performance of the final pipeline on their cohorts. The odds ratios for each condition that are reported on the clinical reports come from these cohorts rather than the eMERGE cohort for the reasons described above.

### PRS ancestry calibration overview

#### PCA method description

For a PRS, which is a sum of SNP effects (linear weights), the central limit theorem states that the distribution of scores in a homogenous population will tend towards a normal distribution as the number of SNPs becomes large. When two different homogenous populations are randomly mixed, the additive property of the PRS leads the resulting distribution to be similarly normally distributed, with mean and variance depending on the mean and variance of the original homogenous populations^37,38^. We can therefore model the distribution of the PRS as being normally distributed, with mean and variance being functions of genetic ancestry. Practically, we implement this as

1 $${\mathrm{{\it{PRS}}_{{raw}}}}=N(\mu ,{\sigma }^{2})$$

2 $$\mu ={\alpha }_{0}+\sum {\alpha }_{i}{\mathrm{P{C}}}_{i}$$

3 $$\begin{array}{l}{\sigma }^{2}=\exp \left({\beta }_{0}+\sum {\beta }_{i}{\mathrm{PC}}_{i}\right),\end{array}$$

with genetic ancestry being represented by projection into principal component (PC) space^39^. The *α* and *β* parameters are found by jointly fitting them to a cohort of training data. This fit is performed by minimizing the negative log likelihood:

4 $$-\log L=\sum _{i}\log {\sigma }_{i}+1/2{\left(\frac{{{\mathrm{pr}}}{\mathrm{{s}}}_{i}-{\mu }_{i}}{{\sigma }_{i}}\right)}^{2},$$

where *i* runs over the individuals in the training cohort, prs_*i*_ is the *i*th individual’s raw PRS, and *μ*_*i*_ and *σ*_*i*_ are calculated using equations (2) and (3) above by projecting the *i*th individual into PC space. Note that, due to the simplicity of the model, overfitting is unlikely to be a problem, and so no regularization or other overfitting avoidance technique is implemented. An individual’s PRS *z*-score can then be calculated as

5 $$z-{{\mathrm{score}}}=\left(\frac{{{\mathrm{prs}}}-\mu }{\sigma }\right),$$

where *μ* and *σ* have again been calculated based on the specific individual’s projection into PC space. In this way, once the model has been trained, the *z*-score calculation is fully defined by the fitted model parameters, and *z*-scores can be calculated without needing additional access to the original training cohort.

#### Generating trained models from All of Us data

Generating the trained models consisted of three steps: (1) selecting the training cohort; (2) imputation of the training cohort; and (3) training the models on the training cohort. A test cohort was also generated to test the performance of the training.

Ancestry-balanced training and test cohorts were generated by subsampling from an initial cohort of around 100,000 All of Us samples. For the purposes of balancing the cohort, each sample was assigned to one of the five 1KG super populations. Principal component analysis was first performed on a random subset of 20,000 samples. The 1KG samples were projected onto these principal components, and a support vector machine was trained on 1KG to predict ancestry. The support vector machine was then used to assign 108,000 AoU samples to one of the five 1KG super populations. A balanced training cohort was selected based on these predicted ancestries, and principal components were recalculated using this balanced training cohort. A similarly balanced test cohort was selected based on ancestries estimated from projection on the training set PCs. The resulting breakdown of the cohorts by estimated ancestry is shown in Extended Data Table 3.

**Extended Data Fig. 2:**
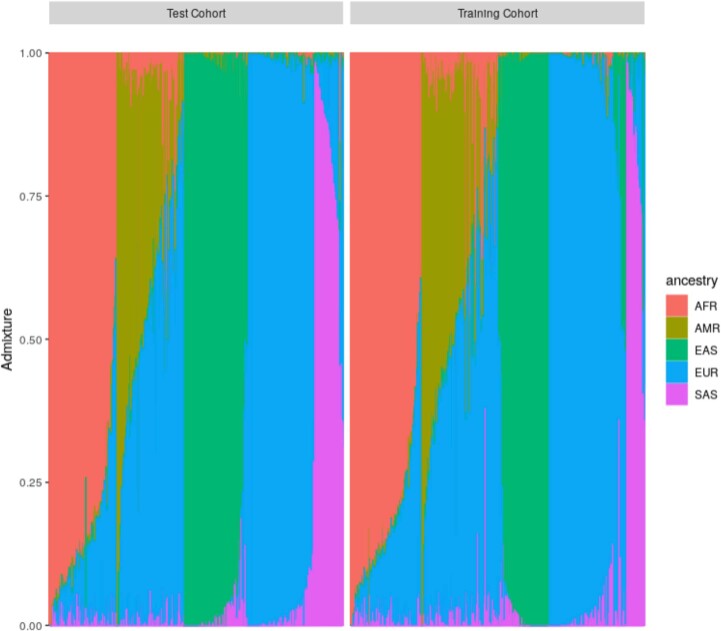
Representation of the genetic ancestry admixture composition of both the Test and Training cohorts. The x-axis represents individuals within the cohorts and the color-coding highlights the proportion of genetic admixture observed.

Both the training and testing cohorts include a number of individuals with highly admixed ancestry. Admixture was quantified using the tool Admixture^40^ with five ancestral populations. The resulting admixtures of each cohort are shown in Extended Data Fig. 2, and the most common admixed ancestries in each cohort are summarized in Extended Data Table 4.

Each cohort was imputed using the imputation pipeline described above, with 1KG as the reference panel. By keeping the imputation pipeline identical to the pipeline used for the eMERGE dataset, and because the AoU dataset uses the same GDA array as the eMERGE dataset, any potential biases resulting from differing data production and processing methods were removed. The training cohort was scored for each of the ten conditions, and model parameters were fit by minimizing the negative log likelihood as described. The test cohort was then used to evaluate the generalizability of these model parameters.

#### Performance on test cohort

**Extended Data Fig. 3:**
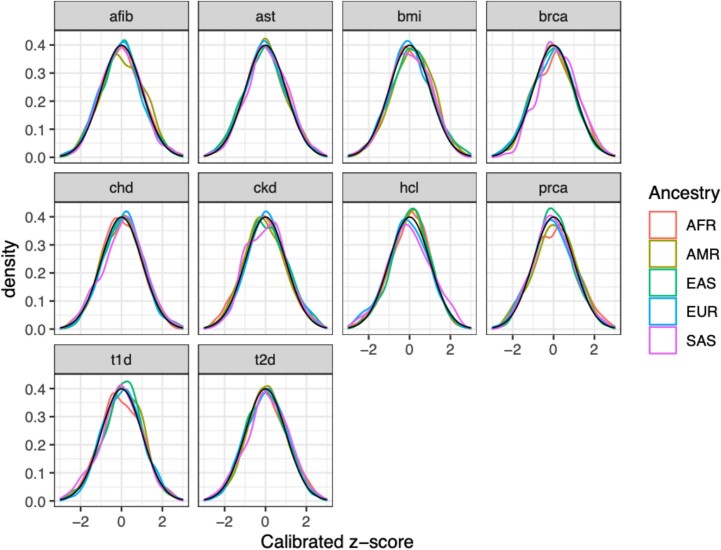
Calibrated z-scores. The distributions of calibrated z-scores in the test cohort when the training cohort parameters are applied.

Extended Data Fig. 3 illustrates the distribution of calibrated *z*-scores in the test cohort using the parameters fit in the training cohort. As can be seen, all ancestries show the intended standard normal distribution of calibrated scores.

**Extended Data Fig. 4:**
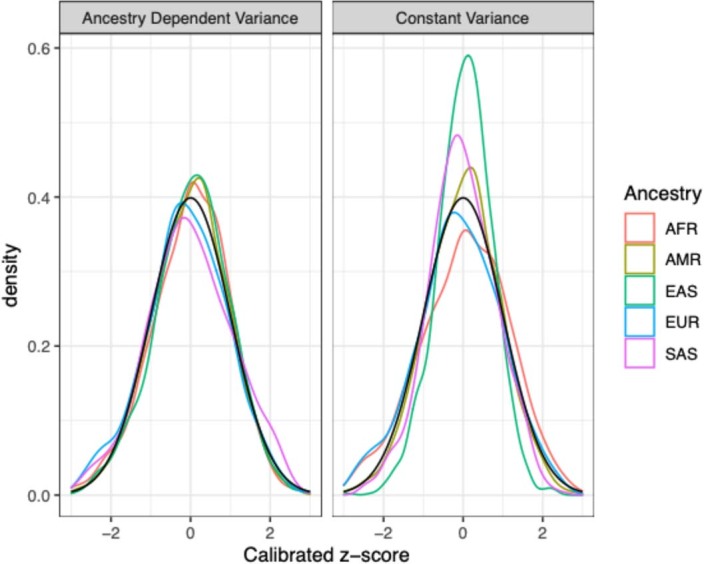
Hypercholesterolemia PRS calibrated z-scores of training cohort. Note the improvement when an ancestry dependent variance is used over a constant variance method.

One of the main improvements of this method over previous methods is the inclusion of an ancestry-dependent variance in addition to the ancestry-dependent mean. The importance of this is shown for the hypercholesterolemia PRS in Extended Data Fig. 4. The variance of this score differs significantly across ancestries, so that a method that only fits the mean of the distribution as ancestry dependent can result in *z*-score distributions that have been attenuated towards zero or expanded away from zero for some ancestries. By also treating variance as ancestry dependent, this method results in *z*-score distributions that are more standardized across ancestries.

**Extended Data Fig. 5:**
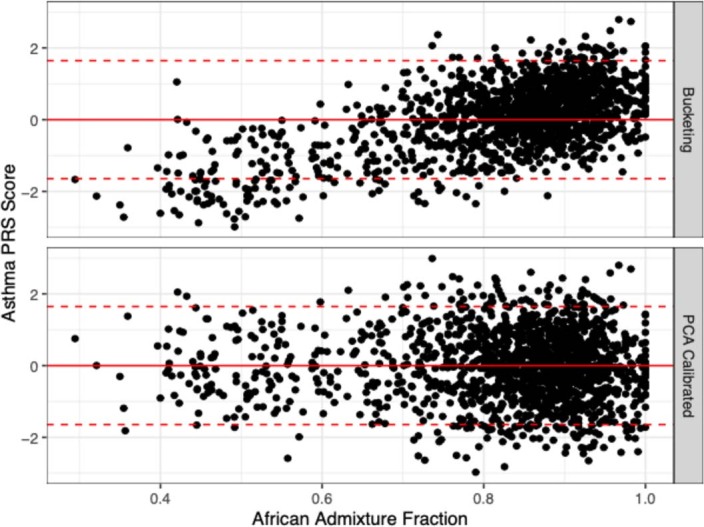
PRS z-score as a function of African Admixture Fraction, for individuals of African ancestry. In the ‘Bucketing’ method, a z-score is calculated by comparing to the mean and variance of all individuals of African ancestry in the cohort. The ‘PCA Calibrated’ method is the method described above. Note the dependence on admixture fraction in the ‘Bucketing’ method, which has been removed in the ‘PCA Calibrated’ method.

In addition to improving calibration across ancestries, this method can improve calibration within ancestries, particularly for highly admixed individuals. An example of this can be seen in Extended Data Fig. 5. Because no ancestry group is homogenous, when individuals are compared directly to other individuals in their assigned population group, a dependence between admixture fraction and PRS can result. This dependence is removed by the described PCA calibration method, and the resulting calibrated PRSs are independent of admixture fraction.

### Reporting summary

Further information on the research design is available in the Nature Portfolio Reporting Summary linked to this article.

## Online content

Any methods, additional references, Nature Portfolio reporting summaries, source data, extended data, supplementary information, acknowledgements, peer review information; details of author contributions and competing interests; and statements of data and code availability are available at 10.1038/s41591-024-02796-z.

### Supplementary information

Supplementary Information Sample clinical report and list of consortia members.

Reporting Summary

Supplementary Table 1 Supplementary Tables 1–3 (tabs in a single worksheet).

## Figures and Tables

**Extended Data Table 1:**
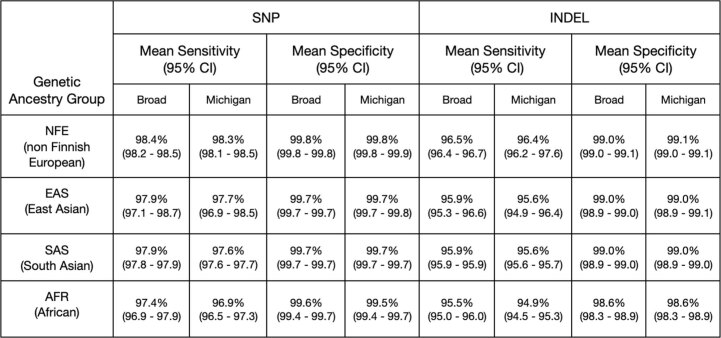
Sensitivity and specificity of SNP and INDEL imputation from the Broad Imputation Pipeline (Broad) and the Michigan Imputation Pipeline (Michigan) with the curated 1000Genomes reference panel when compared to matched whole genome data for 42 AoU v1 samples

**Extended Data Table 2:**
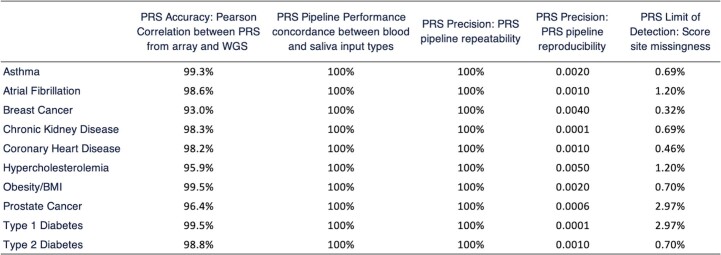
Validation measures summary

PRS pipeline accuracy is assessed as the Pearson correlation between scores derived from PCR-free WGS and those derived from imputed genotyping data (GDA) in 70 specimens. Pearson correlation shown is the mean correlation across all ancestry groups tested.PRS pipeline performance as a function of sample input was assessed by comparing the scores from 20 matched blood and saliva pairs. PRS pipeline precision (repeatability) is the measure of concordance in PRS scores calculated from 70 specimens, run through the pipeline 10 times over the course of two weeks. PRS pipeline precision (reproducibility) is assessed using three samples, each run 6 times end-to-end and then compared in a pairwise manner and represented as the z-score standard deviation is used as a measure of variability. PRS Limit of Detection: score site missingness is the percentage of the original score sites that are missing from the final imputed dataset.

**Extended Data Table 3:**
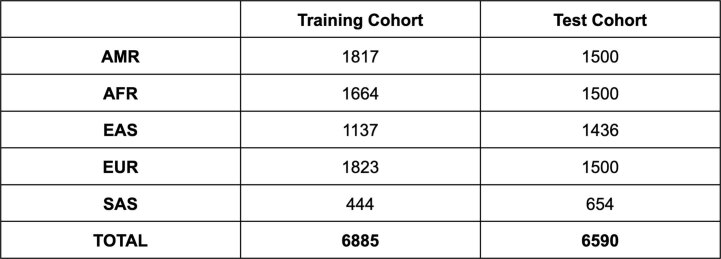
The numbers of participant data files in the training and test cohorts created within the All of Us dataset for the purposes of generating the ancestry calibration model parameters

**Extended Data Table 4:**
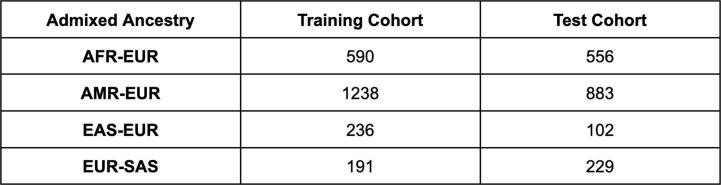
Admixed ancestries are defined as ancestries for which an individual’s admixture fraction is greater than 20%. For example, an individual who is indicated by admixture to be 45% AFR, 37% EUR, 12 % AMR, 5% EAS, 1% SAS would be included in the AFR-EUR row of this table

## Data Availability

Underlying data used to verify the performance of the PRS pipeline are available in dbGaP https://www.ncbi.nlm.nih.gov/projects/gap/cgi-bin/study.cgi?study_id=phs001584.v1.p1. De-identified data relating to trial participants will be available through dbGaP (https://www.ncbi.nlm.nih.gov/gap/) access and the AnVIL platform (https://anvil.terra.bio/) as an interim analysis in 2024 and final dataset at the end of the study, expected in 2026. Information (sites and weights) on the implemented scores can be found at https://github.com/broadinstitute/eMERGE-implemented-PRS-models-Lennon-et-al and also on the UCSC browser https://genome.ucsc.edu/s/Max/emerge. Additionally, PGS Catalog IDs for most of the implemented scores are indicated in Supplementary Table 3.

## Code availability

Codes used in this work to create and operate the imputation and PRS pipelines are hosted at https://github.com/broadinstitute/palantir-workflows/tree/main/ImputationPipeline. Code for the PRS ancestry calibration can also be found in the AoU demonstration workspace https://workbench.researchallofus.org/workspaces/aou-rw-bef5bf62/demopolygenicriskscoregeneticancestrycalibration/data (open access but researcher registration required).
