# Supplementary material for: Selection, optimization and validation of ten chronic disease polygenic risk scores for clinical implementation in diverse US populations
Source: Nat Med. 2024 Feb 19;30(2):480–7. doi: 10.1038/s41591-024-02796-z (PMC10878968; doi:10.1038/s41591-024-02796-z)
Supplement: Supplementary file 1 — Sample clinical report and list of consortia members. [file 41591_2024_2796_MOESM1_ESM.pdf]

nature medicine

Article https://doi.org/10.1038/s41591-024-02796-z

Selection, optimization and validation of ten chronic disease polygenic risk scores for clinical implementation in diverse US populations

In the format provided by the authors and unedited

eMERGE Polygenic Risk Report

PATIENT INFORMATION		REFERRING PROVIDER		SPECIMEN	
Patient Name:	TEST_FN TEST_LN	Provider Name:	TEST_PROVIDER	Report Type:	Final
Date of Birth:	11/01/1956	Referring Facility:	Vanderbilt University Medical Center	Collected:	01/15/2021
Sample ID:	TEST_SAMPLE	Test Performed:	Polygenic Risk Evaluation	Received:	01/01/2021
Patient ID:	INTERNAL_TEST_SAMPLE	Indication:	N/A	Report Date:	03/23/2023
Accession ID:	test_high_risk			Material Type:	DNA
Site Sample ID:	TEST_SAMPLE			Material Source:	Blood

Results Summary

In this patient the Polygenic Risk Score for the following condition(s) was determined to be **HIGH***:

asthma

*See detailed results for a description for how this risk was determined.

Detailed Results

This patient met the threshold for HIGH POLYGENIC RISK for the following condition(s):

Condition: asthma

1. A high polygenic risk score for asthma was found in this individual. A high polygenic risk score is associated with up to 2 times increased risk for developing asthma relative to a person not in the high risk category. The data is based on pediatric populations of European, African, and Hispanic/Latino descent. Information is insufficient or not available for populations of other descent.
2. Factors including monogenic disease risk, family history, and other clinical measures can have an impact on the individuals overall (absolute) risk and should be considered.
3. This participant was tested as part of the Electronic Medical Records and Genomics (eMERGE) Genomic Risk Assessment and Management Study. The participant's integrated Genome Informed Risk Assessment (GIRA) report will be generated which will incorporate the results from this report as well as family history and monogenic risk status, if available.

Other Results

Specific polygenic risk score details for coronary heart disease and breast cancer

The specific polygenic risk score value (expressed as a z-score) for coronary heart disease and breast cancer are being reported for inclusion in absolute risk models that a healthcare provider may choose to use.

Condition:	Test z-score:
breast cancer	N/A
coronary heart disease	1.00

Limitations

- A polygenic score is neither deterministic nor diagnostic. Some people with a 'high risk' polygenic score will never develop the disease while others with a 'not high risk' polygenic score still have a risk of developing the disease that is equal to the general population. Therefore, this test is not intended to diagnose a disease or to make surgical or pharmacological intervention decisions. This test does not tell a patient anything about their current state of health and should not substitute for regular visits to the doctor. Any diagnostic or treatment decisions should be based on additional testing and/or other information that is managed by a healthcare provider.
- This test will detect genetic variants that are predefined. This test does not evaluate or report on all possible genetic variation related to a given disease. It will not detect novel or rare genetic variants and will not rule out the presence of these additional genetic variants related to a disease.
- The predefined list of genetic variants tested in this assay may be different from another institution or company, therefore genetic risk calculations and polygenic scores may differ if compared across different institutions or companies.
- The odds ratio (OR) listed for each condition does not take into account other factors that may play a role in a patient's overall risk of developing a disease (e.g family history or monogenic risk of developing the disease, or environmental and lifestyle risk factors).
- Receiving genetic test results may induce patient anxiety. Patients should speak to their doctor or healthcare provider regarding these test results and potential implications for their health and lifestyle decisions. If you have questions regarding your test results, you can contact the eMERGE clinical study team using the instructions provided to you during the consent process.
- Although the polygenic score has been developed to maximize the ability to predict risk in all ancestries, the availability of population reference data means that the score is currently most accurate for those with European ancestry. Due to this population limitation, as well as assay performance and processing issues, some patients may not receive a polygenic risk calculation for every condition listed. A result of "Not Resulted" for one condition does not impact the reliability of the risk polygenic score of other conditions.

Methodology

A genotyping microarray (the Global Diversity Array from Illumina, Inc.) was used to call single nucleotide variants (SNVs) at ~1.8 million sites in the genome. Only arrays meeting the QC criteria of >98% Call Rate were passed through to the downstream steps. Millions more SNVs in the sample were determined through imputation using data from the 1000 Genomes Project as a reference panel (Khera et al. 2018). Statistical association of SNVs and each condition listed were previously determined through examination of thousands of patient records and genomic data. The relative contribution of the set of SNVs associated with each condition were combined to provide a score. The score was further adjusted to account for the frequency of SNVs in different ancestry populations. The adjusted score was then represented as odds ratio (OR) and 95% confidence interval (CI) compared to a reference population.

The test is validated to determine polygenic risk for 10 conditions: asthma, atrial fibrillation, breast cancer, chronic kidney disease, coronary heart disease, hypercholesterolemia, obesity, prostate cancer, type 1 diabetes, and type 2 diabetes. Only conditions that meet the condition-specific criteria for high polygenic risk are reported as such by this test. In patients younger than 18 years of age, ONLY the following conditions are examined for risk: asthma, obesity, type 1 diabetes, and type 2 diabetes. Breast cancer is only assessed for individuals who elected their sex at birth as female and prostate cancer is only assessed for individuals who have elected their sex at birth as male. The methodologies used to determine the risk criteria for each condition are listed below:

asthma polygenic risk status was determined based on a method that scores 985,837 sites in the genome. A Bayesian regression framework method was applied using the Trans-National Asthma Genetic Consortium (TAGC) GWAS to derive a multi-ancestral PRS score (PMID: 29273806). In a multiethnic study, individuals in the top 5% of the risk percentile had an increased risk of developing asthma. Values within the top 5% of this polygenic risk score are associated with a 1.95 odds ratio (OR) in pediatric populations of European descent at a 95% CI [1.43-2.65], 1.83 OR in pediatric populations of African descent at a 95% CI [1.24-2.70], and 3.12 OR in pediatric populations of Hispanic/Latino descent at 95% CI [1.32-7.44]. Information is insufficient or not available for populations of other descent.

atrial fibrillation polygenic risk status was determined based on a method developed by Nielsen et al. (PMID: 30061737) that scores 161 sites in the genome. In a multiethnic study, individuals in the top 3% of the risk percentile exhibited an increased risk of developing atrial fibrillation. Values within the top 3% of this polygenic risk score are associated with a 2.32 OR in populations of European descent at a 95% CI [2.07-2.61], 2.19 OR in populations of African descent at a 95% CI [1.38-3.38], and 2.27 OR in populations of Hispanic/Latino descent at a 95% CI [1.09-4.50]. Information is insufficient or not available for populations of other descent.

breast cancer polygenic risk status was determined based on a method developed by Mavaddat et al. (PMID: 30554720) that scores 308 sites in the genome. In a multiethnic study, individuals who fell in the top 5% of the risk percentile exhibited an increased risk of developing breast cancer. Values within the top 5% of this polygenic risk score are associated with a 2.47 OR in populations of European descent at a 95% CI [2.20 - 2.77] (PMID: 30554720), 1.61 OR in populations of African descent at a 95% CI [1.38-1.87] (PMID: 33769540), 2.05 OR in populations of Hispanic/Latino descent at a 95% CI [1.10-3.83] (PMID: 34347061), and 2.22 OR in populations of Asian descent at a 95% CI [1.99-2.47] (PMID: 32737321). Information is insufficient or not available for populations of other descent.

chronic kidney disease polygenic risk status was determined based on a study of renal function by Wuttke et al. (PMID: 31152163) and includes 471,316 sites in the genome. In a multiethnic validation study, individuals who fell in the top 2% of the risk percentile exhibited an increased risk of developing chronic kidney disease. Values within the top 2% of this polygenic risk score are associated with a 3.60 OR in populations of European descent at a 95% CI [3.11-4.17], 2.66 OR in populations of African descent at a 95% CI [2.01-3.51], 4.93 OR in populations of Hispanic/Latino descent at a 95% CI [2.46-9.89], and 2.81 OR in populations of Asian descent at a 95% CI [1.91-7.59]. Information is insufficient or not available for populations of other descent.

coronary heart disease polygenic risk status was determined based on a method that scores 458,384 sites in the genome. In a multiethnic study, individuals in the top 5% of the risk percentile exhibited an increased risk of developing coronary heart disease. Values within the top 5% of this polygenic risk score are associated with a relative risk of 2.30 in populations of European descent at a 95% CI [2.07-2.56], 1.68 in populations of African descent at a 95% CI 1.39-2.032], and 2.16 in populations of Hispanic/Latino descent at a 95% CI [1.47-3.19]. For Asian populations, the relative risk is similar to that in European populations. Information is insufficient or not available for populations of other descent.

hypercholesterolemia polygenic risk status was determined based on a method that scores 9,009 sites in the genome. In a multiethnic study, individuals who fell in the top 3% of the risk percentile exhibited an increased risk of developing hypercholesterolemia. Values within the top 3% of this polygenic risk score are associated with a 4.16 OR in populations of European descent at a 95% CI [2.59-6.44], 3.16 OR in populations of African descent at a 95% CI [1.92-5.01], 4.02 OR in populations of Hispanic/Latino descent at a 95% CI [2.72-5.83], and 3.75 OR in populations of Asian descent at a 95% CI [3.15-4.42]. Information is insufficient or not available for populations of other descent.

obesity polygenic risk status was determined based on a method that scores 1,217,710 sites in the genome. In a multiethnic study, individuals who fell in the top 3% of the risk percentile exhibited an increased risk above baseline of developing obesity. Values within the top 3% of this polygenic risk score are associated with a 4.08 OR in populations of European descent at a 95% CI [3.02-5.52], 2.54 OR in populations of African descent at a 95% CI [1.66-3.98], 2.33 OR in populations of Hispanic/Latino descent at a 95% CI [1.64-3.31], and 5.73 OR in populations of Asian descent at a 95% CI [2.28-14.57]. Information is insufficient or not available for populations of other descent.

prostate cancer polygenic risk status was determined based on a method developed by Conti et al. (PMID: 33398198) that scores 264 sites in the genome. In this multiethnic study of individuals of African, European, Asian, and Hispanic/Latino descent, individuals who fell in the top 10% of the risk percentile exhibited an increased risk of developing prostate cancer. Values within the top 10% of this polygenic risk score are associated with a 3.67 OR in populations of European descent at a 95% CI [3.57-3.76] and a 2.95 OR in populations of African descent at a 95% CI [2.60-3.30]. Information is insufficient or not available for populations of other descent.

type 1 diabetes polygenic risk status was determined based on a method developed by Sharp et al. (PMID: 30655379) that scores 71 sites in the genome. In a separate multiethnic study, individuals who fell in the top 3% of the risk percentile exhibited an increased risk of developing type 1 diabetes. Values within the top 3% of this polygenic risk score are associated with a 12.97 OR in populations of European descent at a 95% CI [7.29-20.40] and 20.45 OR in populations of African descent at a 95% CI [10.77-38.83]. Information is insufficient or not available for populations of other descent.

type 2 diabetes polygenic risk status was determined based on a method developed by Ge et al. (medRxiv 2021.09.11.21263413) that scores 1,259,754 sites in the genome. In a multiethnic study, individuals who fell in the top 2% of the risk percentile exhibited an increased risk of developing type 2 diabetes. Values within the top 2% of this polygenic risk score are associated with a 4.21 OR in populations of European descent at a 95% CI [3.66-4.84], 2.55 OR in populations of African descent at a 95% CI [2.09-3.11], 4.58 OR in populations of Asian descent at a 95% CI [4.00-5.23], and 6.87 OR for Hispanic/Latino populations at a 95% CI [3.11, 15.15]. Information is insufficient or not available for populations of other descent.

Regulatory Disclosures

This test was performed by the Clinical Research Sequencing Platform (CRSP), LLC. (320 Charles Street, Cambridge, MA 02141; CLIA: 22D2055652). CRSP, LLC. is authorized under the Clinical Laboratory Improvement Amendments (CLIA) to develop and perform high complexity clinical laboratory testing. This test was developed and its performance characteristics determined by CRSP, LLC. The U.S. Food and Drug Administration (FDA) has not approved or cleared this test; however, FDA clearance or approval is not currently required for clinical use.

References

Conti DV, Darst BF, Moss LC, et al. Trans-ancestry genome-wide association meta-analysis of prostate cancer identifies new susceptibility loci and informs genetic risk prediction. Nat Genet. 2021 Jan;53(1):65-75. doi: 10.1038/s41588-020-00748-0. Epub 2021 Jan 4. Erratum in: Nat Genet. 2021 Jan 20: PMID: 33398198.

Demenais F, Margaritte-Jeannin P, Barnes KC, Cookson WOC, Altmuller J, Ang W, et al. Multiancestry association study identifies new asthma risk loci that colocalize with immune-cell enhancer marks. Nat Genet. 2018;50(1):42-53. Epub 2017/12/24. doi: 10.1038/s41588-017-0014-7. PubMed PMID: 29273806; PMCID: PMC5901974.

Du Z, Guimin G, Adedokun B, et al. Evaluating polygenic risk scores for breast cancer in women of African ancestry. J Natl Cancer Inst. 2021 Sep 4;113(0):1168-1176. doi: 10.1093/jnci/djab050. PMID: 33769540.

Ge T, Patka A, Srinivasasainagendra V, et al. Validation of a trans-ancestry polygenic risk score for type 2 diabetes in diverse populations. medRxiv 2021.09.11.21263413; https://www.medrxiv.org/content/10.1101/2021.09.11.21263413v1.

Ho WK, Tan MM, Mavaddat N, et al. European polygenic risk scores for prediction of breast cancer shows similar performance in Asian women. Nat Commun. 2020 July 31;11(1):3833. doi: 10.1038/s41467-020-17680-w. PMID: 32737321.

Liu C, Zeinomar N, Chung WK, et al. Generalizability of polygenic risk scores for breast cancer among women with European, African, and Latinx ancestry. JAMA Netw Open. 2021 Aug 2;4(8):e2119084. doi: 10.1001/jamanetworkopen. 2021.19084. PMID 34347061.

Mavaddat N, Michailidou K, Dennis J, et al. Polygenic Risk Scores for Prediction of Breast Cancer and Breast Cancer Subtypes. Am J Hum Genet. 2019 Jan 3;104(1):21-34. doi: 10.1016/j.ajhg.2018.11.002. PMID: 30554720.

Nielsen JB, Thorolfsdottir RB, Fritsche LG, et al. Biobank-driven genomic discovery yields new insight into atrial fibrillation biology. Nat Genet. 2018 Sep;50(9):1234-1239. doi: 10.1038/s41588-018-0171-3. PMID: 30061737.

Ruan Y, Lin YF, Feng YCA, Chen CY, Lam M, Guo Z, Stanley Global Asia Initiatives, He L, Sawa A, Martin AR, Qin S, Huang H, Ge T. Improving Polygenic Prediction in Ancestrally Diverse Populations. medRxiv 2020.12.27.20248738; doi: https://doi.org/10.1101/2020.12.27.20248738.

Sharp SA, Rich SS, Wood AR, et al. Development and Standardization of an Improved Type 1 Diabetes Genetic Risk Score for Use in Newborn Screening and Incident Diagnosis. Diabetes Care. 2019 Feb;42(2):200-207. doi: 10.2337/dc18-1785. PMID: 30655379.

Wuttke M, Li Y, Li M, Sieber KB, et al. A catalog of genetic loci associated with kidney function from analyses of a million individuals. Nat Genet. 2019 Jun;51(6):957-972. doi: 10.1038/s41588-019-0407-x. PMID: 31152163.

Testing performed under the direction of Heidi L. Rehm, PhD, FACMG

Results reviewed and approved for release by:

Consortia

The GIANT Consortium

Lo.c Yengo[1], Sailaja Vedantam[2,3], Eirini Marouli[4], Julia Sidorenko[1], Eric Bartell[2,3,5], Saori Sakaue[3,6,7,8], Marielisa Graff[9], Anders U. Eliasen[10,11], Yunxuan Jiang[12], Sridharan Raghavan[13,14], Jenkai Miao[2,3], Joshua D. Arias[15], Sarah E. Graham[16], Ronen E. Mukamel[3,17,18], Cassandra N. Spracklen[19,20], Xianyong Yin[21], Shyh-Huei Chen[22], Teresa Ferreira[23], Heather H. Highland[9], Yingjie Ji[24], Tugce Karaderi[25,26], Kuang Lin[27], Kreete Lüll[28], Deborah E. Malden[27], Carolina Medina-Gomez[29], Moara Machado[15], Amy Moore[30], Sina Rüeger[31,32], Xueling Sim[33], Scott Vrieze[34], Tarunveer S. Ahluwalia[35,36], Masato Akiyama[6,37], Matthew A. Allison[38], Marcus Alvarez[39], Mette K. Andersen[40], Alireza Ani[41,42], Vivek Appadurai[43], Liubov Arbeeva[44], Seema Bhaskar[45], Lawrence F. Bielak[46], Sailalitha Bollepalli[47], Lori L. Bonnycastle[48], Jette Bork-Jensen[40], Jonathan P. Bradfield[49,50], Yuki Bradford[51], Peter S. Braund[52,53], Jennifer A. Brody[54], Kristoffer S. Burgdorf[55,56], Brian E. Cade[5,57], Hui Cai[58], Qiuyin Cai[58], Archie Campbell[59], Marisa Ca.adas-Garre[60], Eulalia Catamo[61], Jin-Fang Chai[33], Xiaoran Chai[62,63], Li-Ching Chang[64], Yi-Cheng Chang[64,65,66], Chien-Hsiun Chen[64], Alessandra Chesi[67,68], Seung Hoan Choi[69], Ren-Hua Chung[70], Massimiliano Cocca[61], Maria Pina Concas[61], Christian Couture[71], Gabriel Cuellar-Partida[12,72], Rebecca Danning[73], E. Warwick Daw[74], Frauke Degenhard[75], Graciela E. Delgado[76], Alessandro Delitala[77], Ayse Demirkan[78,79], Xuan Deng[80], Poornima Devineni[81], Alexander Dietl[82,83], Maria Dimitriou[84], Latchezar Dimitrov[85], Rajkumar Dorajoo[86,87], Arif B. Ekici[88], Jorgen E. Engmann[89], Zammy Fairhurst-Hunter[27], Aliki-Eleni Farmaki[84], Jessica D. Faul[90], Juan-Carlos Fernandez-Lopez[91], Lukas Forer[92], Margherita Francescatto[93], Sandra Freitag-Wolf[94], Christian Fuchsberger[95], Tessel E. Galesloot[96], Yan Gao[97], Zishan Gao[98,99,100], Frank Geller[101], Olga Giannakopoulou[4], Franco Giulianini[73], Anette P. Gjesing[40], Anuj Goel[26,102], Scott D. Gordon[103], Mathias Gorski[82], Jakob Grove[104,105,106], Xiuqing Guo[107], Stefan Gustafsson[108], Jeffrey Haessler[109], Thomas F. Hansen[43,56,110], Aki S. Havulinna[47,111], Simon J. Haworth[112,113], Jing He[58], Nancy Heard-Costa[114,115], Prashantha Hebbar[116], George Hindy[3,117], Yuk-Lam A. Ho[118], Edith Hofer[119,120], Elizabeth Holliday[121], Katrin Horn[122,123], Whitney E. Hornsby[16], Jouke-Jan Hottenga[124], Hongyan Huang[125], Jie Huang[126,127], Alicia Huerta-Chagoya[128,129,130], Jennifer E. Huffman[118], Yi-Jen Hung[131], Shaofeng Huo[132], Mi Yeong Hwang[133], Hiroyuki Iha[134], Daisuke D. Ikeda[134], Masato Isono[135], Anne U. Jackson[21], Susanne J.ger[136,137], Iris E. Jansen[138,139], Ingegerd Johansson[140,141], Jost B. Jonas[142,143,144,145], Anna Jonsson[40], Torben J.rgensen[146,147], Ioanna-Panagiota Kalafati[84], Masahiro Kanai[3,6,7], Stavroula Kanoni[4], Line L. K.rhus[146], Anuradhani Kasturiratne[148], Tomohiro Katsuya[149], Takahisa Kawaguchi[150], Rachel L. Kember[151], Katherine A. Kentistou[152,153], Han-Na Kim[154,155], Young Jin Kim[133], Marcus E. Kleber[76,156], Maria J. Knol[78], Azra Kurbasic[157], Marie Lauzon[107], Phuong Le[158,159], Rodney Lea[160], Jong-Young Lee[161], Hampton L. Leonard[162,163,164], Shengchao A. Li[15,165], Xiaohui Li[107], Xiaoyin Li[166,496], Jingjing Liang[166], Honghuang Lin[167], Shih-Yi Lin[168], Jun Liu[27,78], Xueping Liu[101], Ken Sin Lo[169], Jirong Long[58], Laura Lores-Motta[170], Jian'an Luan[171], Valeriya Lyssenko[172,173], Leo-Pekka Lyytik.inen[174,175,176], Anubha Mahajan[26,498], Vasiliki Mamakou[177], Massimo Mangino[178,179], Ani Manichaikul[180], Jonathan Marten[181], Manuel Mattheisen[104,182,183], Laven Mavarani[184], Aaron F. McDaid[31,32], Karina Meidtner[136,137], Tori L. Melendez[16], Josep M. Mercader[18,128,185,186], Yuri Milaneschi[187], Jason E. Miller[188,189], Iona Y. Millwood[27,190], Pashupati P. Mishra[174,175], Ruth E. Mitchell[112,191], Line T. M.llehave[146], Anna Morgan[61], Soeren Mucha[192], Matthias Munz[192], Masahiro Nakatochi[193], Christopher P. Nelson[52,53], Maria Nethander[194,195], Chu Won Nho[196], Aneta A. Nielsen[197], Ilja M. Nolte[41], Suraj S. Nongmaithem[45,198], Raymond Noordam[199], Ioanna Ntalla[4],

Teresa Nutile[200] , Anita Pandit[21] , Paraskevi Christofidou[178] , Katri P.rna[28,41] , Marc Pauper[170] , Eva R. B. Petersen[201] , Liselotte V. Petersen[105,202] , Niina Pitk.nen[203,204] , Ozren Polašek[205,206] , Alaitz Poveda[157] , Michael H. Preuss[207,208] , Saiju Pyarajan[5,57,81] , Laura M. Raffield[19] , Hiromi Rakugi[149] , Julia Ramirez[4,209,210] , Asif Rasheed[211] , Dennis Raven[212] , Nigel W. Rayner[26,198,213,214] , Carlos Riveros[215,216] , Rebecca Rohde[9] , Daniela Ruggiero[200,217] , Sanni E. Ruotsalainen[47] , Kathleen A. Ryan[218,219] , Maria Sabater-Lleal[220,221] , Richa Saxena[3,186] , Markus Scholz[122,123] , Anoop Sendamarai[81] , Botong Shen[222] , Jingchunzi Shi[12] , Jae Hun Shin[223] , Carlo Sidore[224] , Colleen M. Sitlani[54] , Roderick C. Slieker[225,226,227] , Roelof A. J. Smit[207,228] ,Albert V. Smith[46,229] , Jennifer A. Smith[46,90] , Laura J. Smyth[60] ,Lorraine Southam[213,230] ,Valgerdur Steinthorsdottir[231] , Liang Sun[132] , Fumihiko Takeuchi[135] ,DivyaSriPriyanka Tallapragada[45,232] , Kent D. Taylor[107] , Bamidele O. Tayo[233] ,Catherine Tcheandjieu[234,235] , Natalie Terzikhan[78] , Paola Tesolin[93] ,Alexander Teumer[236,237] , Elizabeth Theusch[238] ,Deborah J. Thompson[239,240] ,Gudmar Thorleifsson[231] ,Paul R. H. J. Timmers[152,181] ,
Stella Trompet[199,241] , Constance Turman[125] , Simona Vaccargiu[224] , Sander W. van der Laan[242] , Peter J. van der Most[41] , Jan B. van Klinken[243,244,245] , Jessica van Setten[246] , Shefali S. Verma[67] , Niek Verweij[247] , Yogasudha Veturi[51] , Carol A. Wang[215,216] , Chaolong Wang[86,248] , Lihua Wang[74] , Zhe Wang[207] , Helen R. Warren[4,249] , Wen Bin Wei[250] , Ananda R. Wickremasinghe[148] , Matthias Wielscher[251,252] , Kerri L. Wiggins[54] , Bendik S. Winsvold[253,254] , Andrew Wong[255] , Yang Wu[1] , Matthias Wuttke[256,257] , Rui Xia[258] , Tian Xie[41] , Ken Yamamoto[259] , Jingyun Yang[260,261] , Jie Yao[107] , Hannah Young[34] , Noha A. Yousri[262,263] , Lei Yu[260,261] , Lingyao Zeng[264] , Weihua Zhang[265,266] , Xinyuan Zhang[51] , Jing-Hua Zhao[267] , Wei Zhao[46] , Wei Zhou[3,268,269,270] , Martina E. Zimmermann[82] , Magdalena Zoledziewska[224] , Linda S. Adair[271,272] , Hieab H. H. Adams[273,274,275] , Carlos A. Aguilar-Salinas[276,277] , Fahd Al-Mulla[116] , Donna K. Arnett[278], Folkert W. Asselbergs[246,279,280] , Bj.rn Olav .svold[281,282,283] , John Attia[121] , Bernhard Banas[284] , Stefania Bandinelli[285] , David A. Bennett[260,261] , Tobias Bergler[284] , Dwaipayan Bharadwaj[286] , Ginevra Biino[287] , Hans Bisgaard[10] , Eric Boerwinkle[288] , Carsten A. B.ger[284,289,290] , Klaus B.nnelykke[10] , Dorret I. Boomsma[124] , Anders D. B.rglum[104,105,291,292] , Judith B. Borja[293,294] , Claude Bouchard[295] , Donald W. Bowden[85,296] , Ivan Brandslund[297,298] , Ben Brumpton[281,299] , Julie E. Buring[5,73] , Mark J. Caulfield[4,249] , John C. Chambers[265,266,300,301] , Giriraj R. Chandak[45,302] , Stephen J. Chanock[15] , Nish Chaturvedi[255] , Yii-Der Ida Chen[107] , Zhengming Chen[27,190] , Ching-Yu Cheng[62,303] , Ingrid E. Christophersen[304,305] , Marina Ciullo[200,217] , John W. Cole[306,307] , Francis S. Collins[48] , Richard S. Cooper[233] , Miguel Cruz[308] , Francesco Cucca[224,309] , L. Adrienne Cupples[80,115] , Michael J. Cutler[310] , Scott M. Damrauer[51,311,312] , Thomas M. Dantoft[146] , Gert J. de Borst[313] , Lisette C. P. G. M. de Groot[314] , Philip L. De Jager[3,315] , Dominique P. V. de Kleijn[313] , H. Janaka de Silva[148] , George V. Dedoussis[84] , Anneke I. den Hollander[170] , Shufa Du[271,272] , Douglas F. Easton[239,316] , Petra J. M. Elders[317] , A. Heather Eliassen[57,125,318] , Patrick T. Ellinor[69,319,320] , S.lve Elmst.hl[321] , Jeanette Erdmann[192] , Michele K. Evans[222] , Diane Fatkin[322,323,324] , Bjarke Feenstra[101] , Mary F. Feitosa[74] , Luigi Ferrucci[325] , Ian Ford[326] , Myriam Fornage[258,327] , Andre Franke[75] , Paul W. Franks[157,318,328] , Barry I. Freedman[329] , Paolo Gasparini[61,93] , Christian Gieger[99,137] , Giorgia Girotto[61,93] , Michael E. Goddard[330,331] , Yvonne M. Golightly[9,44,332,333] , Clicerio Gonzalez-Villalpando[334] , Penny Gordon-Larsen[271,272] , Harald Grallert[99,137] , Struan F. A. Grant[49,335,336,337] , Niels Grarup[40] , Lyn Griffiths[160] , Vilmundur Gudnason[229,338] , Christopher Haiman[339] , Hakon Hakonarson[49,335,340,341] , Torben Hansen[40] , Catharina A. Hartman[212] , Andrew T. Hattersley[342] , Caroline Hayward[181] , Susan R. Heckbert[343] , Chew-Kiat Heng[344,345] , Christian Hengstenberg[346] , Alex W. Hewitt[347,348,349] , Haretsugu Hishigaki[134] , Carel B. Hoyng[170] , Paul L. Huang[5,320,350] , Wei Huang[351] , Steven C. Hunt[262,352] , Kristian Hveem[281,282] , Elina Hypp.nen[353,354] , William G. Iacono[34] , Sahoko Ichihara[355] , M. Arfan Ikram[78] , Carmen R. Isasi[356] , Rebecca D. Jackson[357] , Marjo-Riitta Jarvelin[251,358,359,360] , Zi-Bing Jin[145,361] ,

Karl-Heinz J.ckel[184] , Peter K. Joshi[153] , Pekka Jousilahti[111] , J. Wouter Jukema[241,362,363] ,
Mika K.h.nen[364,365] , Yoichiro Kamatani[6,366] , Kui Dong Kang[367] , Jaakko Kaprio[47] ,
Sharon L. R. Kardia[46] , Fredrik Karpe[214,368] , Norihiro Kato[135] , Frank Kee[60] ,
Thorsten Kessler[264,369] , Amit V. Khera[3,186] , Chiea Chuen Khor[86] ,
Lambertus A. L. M. Kiemeney[96,370] , Bong-Jo Kim[133] , Eung Kweon Kim[371,372] , Hyung-Lae Kim[373] ,
Paulus Kirchhof[374,375,376,377] , Mika Kivimaki[378] , Woon-Puay Koh[379] , Heikki A. Koistinen[111,380,381] ,
Genovefa D. Kolovou[382] , Jaspal S. Kooner[265,301,383,384] , Charles Kooperberg[109] ,
Anna K.ttgen[256] , Peter Kovacs[385] , Adriaan Kraaijeveld[246] , Peter Kraft[125] , Ronald M. Krauss[238] ,
Meena Kumari[386] , Zoltan Kutalik[31,32] , Markku Laakso[387] , Leslie A. Lange[388] ,
Claudia Langenberg[171,389] , Lenore J. Launer[222] , Loic Le Marchand[390] , Hyejin Lee[391] ,
Nanette R. Lee[293] , Terho Lehtim.ki[174,175] , Huaixing Li[132] , Liming Li[392,393] , Wolfgang Lieb[394] ,
Xu Lin[132,395] , Lars Lind[108] , Allan Linneberg[146,396] , Ching-Ti Liu[80] , Jianjun Liu[86] ,
Markus Loeffler[122,123] , Barry London[397] , Steven A. Lubitz[69,319,320] , Stephen J. Lye[398] ,
David A. Mackey[347,349] , Reedik M.gi[28] , Patrik K. E. Magnusson[399] , Gregory M. Marcus[400] ,
Pedro Marques Vidal[401,402] , Nicholas G. Martin[103] , Winfried M.rz[76,403,404] , Fumihiko Matsuda[150] ,
Robert W. McGarrah[405,406] , Matt McGue[34] , Amy Jayne McKnight[60] , Sarah E. Medland[407] ,
Dan Mellstr.m[194,408] , Andres Metspalu[28] , Braxton D. Mitchell[218,219,409] , Paul Mitchell[410] ,
Dennis O. Mook-Kanamori[228,411] , Andrew D. Morris[412] , Lorelei A. Mucci[125] ,
Patricia B. Munroe[4,249] , Mike A. Nalls[162,163,164] , Saman Nazarian[413] , Amanda E. Nelson[44,414] ,
Matt J. Neville[214,368] , Christopher Newton-Cheh[186,320] , Christopher S. Nielsen[415,416] ,
Markus M. N.then[417] , Claes Ohlsson[194,418] , Albertine J. Oldehinkel[212] , Lorena Orozco[419] ,
Katja Pahkala[203,204,420] , P.ivi Pajukanta[39,421] , Colin N. A. Palmer[422] , Esteban J. Parra[159] ,
Cristian Pattaro[95] , Oluf Pedersen[40] , Craig E. Pennell[215,216] , Brenda W. J. H. Penninx[187] ,
Louis Perusse[71,423] , Annette Peters[100,137,424] , Patricia A. Peyser[46] , David J. Porteous[59] ,
Danielle Posthuma[138] , Chris Power[425] , Peter P. Pramstaller[95] , Michael A. Province[74] ,
Qibin Qi[356] , Jia Qu[361] , Daniel J. Rader[51,426] , Olli T. Raitakari[203,204,427] , Sarju Ralhan[428] ,
Loukianos S. Rallidis[429] , Dabeeru C. Rao[430] , Susan Redline[5,57] , Dermot F. Reilly[431] ,
Alexander P. Reiner[109,432] , Sang Youl Rhee[433] , Paul M. Ridker[5,73] , Michiel Rienstra[247] ,
Samuli Ripatti[3,47,434] , Marylyn D. Ritchie[51] , Dan M. Roden[435] , Frits R. Rosendaal[228] ,
Jerome I. Rotter[107] , Igor Rudan[152] , Femke Rutters[436] , Charumathi Sabanayagam[62,303] ,
Danish Saleheen[211,437] , Veikko Salomaa[111] , Nilesh J. Samani[52,53] ,
Dharambir K. Sanghera[438,439,440,441] , Naveed Sattar[442] , B.rge Schmidt[184] , Helena Schmidt[443] ,
Reinhold Schmidt[119] , Matthias B. Schulze[136,137,444] , Heribert Schunkert[445] , Laura J. Scott[21] ,
Rodney J. Scott[446] , Peter Sever[384] , Eric J. Shiroma[222] , M. Benjamin Shoemaker[447] ,
Xiao-Ou Shu[58] , Eleanor M. Simonsick[325] , Mario Sims[97] , Jai Rup Singh[448] ,
Andrew B. Singleton[162] , Moritz F. Sinner[369,449] , J. Gustav Smith[450,451,452] , Harold Snieder[41] ,
Tim D. Spector[178] , Meir J. Stampfer[57,125,318] , Klaus J. Stark[82] , David P. Strachan[453] ,
Leen M. ‘t Hart[225,226,227,454] , Yasuharu Tabara[150] , Hua Tang[455] , Jean-Claude Tardif[169,456] ,
Thangavel A. Thanaraj[116] , Nicholas J. Timpson[112,191] , Anke T.njes[385] , Angelo Tremblay[71,423] ,
Tiinamaija Tuomi[47,173,457,458] , Jaakko Tuomilehto[111,459,460] , Maria-Teresa Tusi.-Luna[461,462] ,
Andre G. Uitterlinden[29] , Rob M. van Dam[33,463,464] , Pim van der Harst[246,247] ,
Nathalie Van der Velde[29,465] , Cornelia M. van Duijn[27,78] , Natasja M. van Schoor[466] ,
Veronique Vitart[181] , Uwe V.lker[237,467] , Peter Vollenweider[401,402] , Henry V.lzke[236,237] ,
Niels H. Wacher-Rodarte[468] , Mark Walker[469] , Ya Xing Wang[145] , Nicholas J. Wareham[171] ,
Richard M. Watanabe[470,471,472] , Hugh Watkins[26,102] , David R. Weir[90] , Thomas M. Werge[43,396,473] ,
Elisabeth Widen[47] , Lynne R. Wilkens[390] , Gonneke Willemsen[124] , Walter C. Willett[125,318] ,
James F. Wilson[152,181] , Tien-Yin Wong[62,303] , Jeong-Taek Woo[433] , Alan F. Wright[181] ,
Jer-Yuarn Wu[64,474] , Huichun Xu[218,219] , Chittaranjan S. Yajnik[475] , Mitsuhiro Yokota[476] ,
Jian-Min Yuan[477,478] , Eleftheria Zeggini[213,230,479] , Babette S. Zemel[51,68,337,340] , Wei Zheng[58] ,

Xiaofeng Zhu[166] , Joseph M. Zmuda[478] , Alan B. Zonderman[222] , John-Anker Zwart[253,480] , 23andMe Research Team * , VA Million Veteran Program * , DiscovEHR (DiscovEHR and MyCode Community Health Initiative) * , eMERGE (Electronic Medical Records and Genomics Network) * , Lifelines Cohort Study * , The PRACTICAL Consortium * , Understanding Society Scientific Group * , Daniel I. Chasman[5,73] , Yoon Shin Cho[223] , Iris M. Heid[82] , Mark I. McCarthy[26,214,497] , Maggie C. Y. Ng[85,482] , Christopher J. O'Donnell[5,57,483] , Fernando Rivadeneira[29] , Unnur Thorsteinsdottir[231,338] , Yan V. Sun[484,485] , E. Shyong Tai[33,463] , Michael Boehnke[21] , Panos Deloukas[4,486] , Anne E. Justice[9,481] , Cecilia M. Lindgren[3,23,26] , Ruth J. F. Loos[40,207,208,487] , Karen L. Mohlke[19] , Kari E. North[9] , Kari Stefansson[231,338] , Robin G. Walters[27,190] , Thomas W. Winkler[82] , Kristin L. Young[9] , Po-Ru Loh[3,17,18] , Jian Yang[1,488,489] , T.nu Esko[28] , Themistocles L. Assimes[234,235] , Adam Auton[12] , Goncalo R. Abecasis[21] , Cristen J. Willer[16,268,490] , Adam E. Locke[491] , Sonja I. Berndt[15] , Guillaume Lettre[169,456] , Timothy M. Frayling[24] , Yukinori Okada[6,7,492,493,498,499], Andrew R. Wood[24] , Peter M. Visscher[1] & Joel N. Hirschhorn[2,494,495]

[1] Institute for Molecular Bioscience, The University of Queensland, Brisbane, Queensland, Australia. [2] Division of Endocrinology, Boston Children's Hospital, Boston, MA, USA. [3] Program in Medical and Population Genetics, Broad Institute of MIT and Harvard, Cambridge, MA, USA. [4] William Harvey Research Institute, Barts and the London School of Medicine and Dentistry, Queen Mary University of London, London, UK. [5] Harvard Medical School, Boston, MA, USA. [6] Laboratory for Statistical Analysis, RIKEN Center for Integrative Medical Sciences, Yokohama, Japan. [7] Department of Statistical Genetics, Osaka University Graduate School of Medicine, Osaka, Japan. [8] Divisions of Genetics and Rheumatology, Brigham and Women's Hospital and Department of Medicine, Harvard Medical School, Boston, MA, USA. [9] Department of Epidemiology, Gillings School of Global Public Health, University of North Carolina at Chapel Hill, Chapel Hill, NC, USA. [10] COPSAC, Copenhagen Prospective Studies on Asthma in Childhood, Herlev and Gentofte Hospital, University of Copenhagen, Copenhagen, Denmark. [11] Section for Bioinformatics, Department of Health Technology, Technical University of Denmark, Copenhagen, Denmark. [12] 23andMe, Sunnyvale, CA, USA. [13] Department of Veterans Affairs, Eastern Colorado Healthcare System, Aurora, CO, USA. [14] Division of Biomedical Informatics and Personalized Medicine, University of Colorado Anschutz Medical Campus, Aurora, CO, USA. [15] Division of Cancer Epidemiology and Genetics, National Cancer Institute, Rockville, MD, USA. [16] Department of Internal Medicine, Division of Cardiology, University of Michigan, Ann Arbor, MI, USA. [17] Division of Genetics, Department of Medicine, Brigham and Women's Hospital, Boston, MA, USA. [18] Department of Medicine, Harvard Medical School, Boston, MA, USA. [19] Department of Genetics, University of North Carolina at Chapel Hill, Chapel Hill, NC, USA. [20] Department of Biostatistics and Epidemiology, School of Public Health and Health Sciences, University of Massachusetts, Amherst, MA, USA. [21] Department of Biostatistics and Center for Statistical Genetics, University of Michigan School of Public Health, Ann Arbor, MI, USA. [22] Department of Biostatistics and Data Science, Wake Forest School of Medicine, Winston-Salem, NC, USA. [23] Big Data Institute, Li Ka Shing Centre for Health Information and Discovery, University of Oxford, Oxford, UK. [24] Genetics of Complex Traits, College of Medicine and Health, University of Exeter, Exeter, UK. [25] Center for Health Data Science, Faculty of Health and Medical Sciences, University of Copenhagen, Copenhagen, Denmark. [26] Wellcome Centre for Human Genetics, Nuffield Department of Medicine, University of Oxford, Oxford, UK. [27] Nuffield Department of Population Health, University of Oxford, Oxford, UK. [28] Institute of Genomics, Estonian Genome Centre, University of Tartu, Tartu, Estonia. [29] Department of Internal Medicine, Erasmus MC, University Medical

Center Rotterdam, Rotterdam, The Netherlands. 30 Division of Biostatistics and Epidemiology, RTI International, Durham, NC, USA. 31 Center for Primary Care and Public Health, University of Lausanne, Lausanne, Switzerland. 32 Swiss Institute of Bioinformatics, Lausanne, Switzerland. 33 Saw Swee Hock School of Public Health, National University of Singapore and National University Health System, Singapore, Singapore. 34 Department of Psychology, University of Minnesota, Minneapolis, MN, USA. 35 Steno Diabetes Center Copenhagen, Herlev, Denmark. 36 Department of Biology, The Bioinformatics Center, University of Copenhagen, Copenhagen, Denmark. 37 Department of Ophthalmology, Graduate School of Medical Sciences, Kyushu University, Fukuoka, Japan. 38 Department of Family Medicine, University of California, San Diego, La Jolla, CA, USA. 39 Department of Human Genetics, David Geffen School of Medicine at UCLA, Los Angeles, CA, USA. 40 Novo Nordisk Foundation Center for Basic Metabolic Research, Faculty of Health and Medical Sciences, University of Copenhagen, Copenhagen, Denmark. 41 Department of Epidemiology, University of Groningen, University Medical Center Groningen, Groningen, The Netherlands. 42 Department of Bioinformatics, Isfahan University of Medical Sciences, Isfahan, Iran. 43 Institute of Biological Psychiatry, Mental Health Services, Copenhagen University Hospital, Copenhagen, Denmark. 44 Thurston Arthritis Research Center, University of North Carolina at Chapel Hill, Chapel Hill, NC, USA. 45 Genomic Research on Complex diseases (GRC-Group), CSIR-Centre for Cellular and Molecular Biology, Hyderabad, India. 46 Department of Epidemiology, University of Michigan School of Public Health, Ann Arbor, MI, USA. 47 Institute for Molecular Medicine Finland (FIMM), HiLIFE, University of Helsinki, Helsinki, Finland. 48 Molecular Genetics Section, Center for Precision Health Research, National Human Genome Research Institute, National Institutes of Health, Bethesda, MD, USA. 49 Center for Applied Genomics, Children’s Hospital of Philadelphia, Philadelphia, PA, USA. 50 Quantinuum Research, Wayne, PA, USA. 51 Department of Genetics, University of Pennsylvania, Philadelphia, PA, USA. 52 Department of Cardiovascular Sciences, University of Leicester, Leicester, UK. 53 NIHR Leicester Biomedical Research Centre, Glenfield Hospital, Leicester, UK. 54 Cardiovascular Health Research Unit, Department of Medicine, University of Washington, Seattle, WA, USA. 55 Department of Clinical Immunology, Copenhagen University Hospital, Rigshospitalet, Copenhagen, Denmark. 56 NovoNordic Center for Protein Research, Copenhagen University, Copenhagen, Denmark. 57 Department of Medicine, Brigham and Women’s Hospital, Boston, MA, USA. 58 Division of Epidemiology, Department of Medicine, Vanderbilt University Medical Center, Nashville, TN, USA. 59 Centre for Genomic and Experimental Medicine, Institute of Genetics and Cancer, University of Edinburgh, Edinburgh, UK. 60 Centre for Public Health, Queen’s University of Belfast, Belfast, UK. 61 Institute for Maternal and Child Health – IRCCS, Burlo Garofolo, Trieste, Italy. 62 Ocular Epidemiology, Singapore Eye Research Institute, Singapore National Eye Centre, Singapore, Singapore. 63 Department of Ophthalmology, National University of Singapore and National University Health System, Singapore, Singapore. 64 Institute of Biomedical Sciences, Academia Sinica, Taipei, Taiwan. 65 Graduate Institute of Medical Genomics and Proteomics, Medical College, National Taiwan University, Taipei, Taiwan. 66 Department of Internal Medicine, National Taiwan University Hospital, Taipei, Taiwan. 67 Department of Pathology and Laboratory Medicine, University of Pennsylvania, Philadelphia, PA, USA. 68 Center for Spatial and Functional Genomics, Division of Human Genetics, Children’s Hospital of Philadelphia, Philadelphia, PA, USA. 69 Cardiovascular Disease Initiative, Broad Institute of MIT and Harvard, Cambridge, MA, USA. 70 Institute of Population Health Sciences, National Health Research Institutes, Zhunan, Taiwan. 71 Department of Kinesiology, Faculty of Medicine, Universit. Laval, Qu.bec City, Quebec, Canada. 72 Diamantina Institute, The University of Queensland, Brisbane, Queensland, Australia. 73 Division of Preventive Medicine, Brigham and Women’s Hospital, Boston, MA, USA. 74 Division of Statistical Genomics, Department of Genetics,

Washington University School of Medicine, St Louis, MO, USA. [75] Institute of Clinical Molecular Biology, Christian-Albrechts University of Kiel, Kiel, Germany. [76] Vth Department of Medicine, Medical Faculty Mannheim, Heidelberg University, Mannheim, Germany. [77] Dipartimento di Scienze Mediche Chirurgiche e Sperimentali, Universit. degli Studi di Sassari, Sassari, Italy. [78] Department of Epidemiology, Erasmus MC, University Medical Center Rotterdam, Rotterdam, The Netherlands. [79] Section of Statistical Multi-omics, Department of Clinical and Experimental Medicine, University of Surrey, Guildford, UK. [80] Department of Biostatistics, Boston University School of Public Health, Boston, MA, USA. [81] Center for Data and Computational Sciences, VA Boston Healthcare System, Boston, MA, USA. [82] Department of Genetic Epidemiology, University of Regensburg, Regensburg, Germany. [83] Department of Internal Medicine II, University Hospital Regensburg, Regensburg, Germany. [84] Department of Nutrition and Dietetics, School of Health and Education, Harokopio University of Athens, Athens, Greece. [85] Center for Precision Medicine, Wake Forest School of Medicine, Medical Center Boulevard, Winston-Salem, NC, USA. [86] Genome Institute of Singapore, Agency for Science, Technology and Research, Singapore, Singapore. [87] Health Services and Systems Research, Duke-NUS Medical School, Singapore, Singapore. [88] Institute of Human Genetics, Universit.tsklinikum Erlangen, Friedrich-Alexander-Universit.t Erlangen-Nürnberg, Erlangen, Germany. [89] Institute of Cardiovascular Science, Faculty of Population Health, University College London, London, UK. [90] Survey Research Center, Institute for Social Research, University of Michigan, Ann Arbor, MI, USA. [91] Computational Genomics Department, National Institute of Genomic Medicine, Mexico City, Mexico. [92] Institute of Genetic Epidemiology, Medical University of Innsbruck, Innsbruck, Austria. [93] Department of Medicine, Surgery and Health Sciences, University of Trieste, Trieste, Italy. [94] Institute of Medical Informatics and Statistics, Kiel University, Kiel, Germany. [95] Eurac Research, Institute for Biomedicine, Affiliated Institute of the University of Lübeck, Bolzano, Italy. [96] Radboud University Medical Center, Radboud Institute for Health Sciences, Department for Health Evidence, Nijmegen, The Netherlands. [97] Jackson Heart Study, Department of Medicine, University of Mississippi, Jackson, MS, USA. [98] Nanjing University of Chinese Medicine, Nanjing, China. [99] Research Unit of Molecular Epidemiology, Institute of Epidemiology, Helmholtz Zentrum München Research Center for Environmental Health, Neuherberg, Germany. [100] Institute of Epidemiology, Helmholtz Zentrum München Research Center for Environmental Health, Neuherberg, Germany. [101] Department of Epidemiology Research, Statens Serum Institut, Copenhagen, Denmark. [102] Cardiovascular Medicine, Radcliffe Department of Medicine, University of Oxford, John Radcliffe Hospital, Oxford, UK. [103] Genetic Epidemiology, QIMR Berghofer Medical Research Institute, Brisbane, Queensland, Australia. [104] Department of Biomedicine (Human Genetics) and iSEQ Center, Aarhus University, Aarhus, Denmark. [105] The Lundbeck Foundation Initiative for Integrative Psychiatric Research, iPSYCH, Aarhus, Denmark. [106] BiRC—Bioinformatics Research Centre, Aarhus University, Aarhus, Denmark. [107] The Institute for Translational Genomics and Population Sciences, Department of Pediatrics, The Lundquist Institute for Biomedical Innovation at Harbor-UCLA Medical Center, Torrance, CA, USA. [108] Department of Medical Sciences, Uppsala University, Uppsala, Sweden. [109] Division of Public Health Sciences, Fred Hutchinson Cancer Research Center, Seattle, WA, USA. [110] Danish Headache Center, Department of Neurology, Copenhagen University Hospital, Rigshospitalet, Rigshospitalet, Copenhagen, Denmark. [111] Department of Public Health and Welfare, Finnish Institute for Health and Welfare, Helsinki, Finland. [112] MRC Integrative Epidemiology Unit, University of Bristol, Bristol, UK. [113] Bristol Dental School, University of Bristol, Bristol, UK. [114] Department of Neurology, Boston University School of Medicine, Boston, MA, USA. [115] Framingham Heart Study, Framingham, MA, USA. [116] Department of Genetics and Bioinformatics, Dasman Diabetes Institute, Kuwait City, Kuwait. [117] Department of Clinical

Sciences in Malm., Lund University, Malm., Sweden. 118 Veterans Affairs Boston Healthcare System, Boston, MA, USA. 119 Clinical Division of Neurogeriatrics, Department of Neurology, Medical University of Graz, Graz, Austria. 120 Institute for Medical Informatics, Statistics and Documentation, Medical University of Graz, Graz, Austria. 121 School of Medicine and Public Health, University of Newcastle, Callaghan, New South Wales, Australia. 122 Institute for Medical Informatics, Statistics and Epidemiology, University of Leipzig, Medical Faculty, Leipzig, Germany. 123 LIFE Research Center for Civilization Diseases, University of Leipzig, Medical Faculty, Leipzig, Germany. 124 Department of Biological Psychology, Behaviour and Movement Sciences, Vrije Universiteit Amsterdam, Amsterdam, The Netherlands. 125 Department of Epidemiology, Harvard T.H. Chan School of Public Health, Boston, MA, USA. 126 School of Public Health and Emergency Management, Southern University of Science and Technology, Shenzhen, China. 127 Institute for Global Health and Development, Peking University, Beijing, China. 128 Programs in Metabolism and Medical and Population Genetics, Broad Institute of Harvard and MIT, Cambridge, MA, USA. 129 Departamento de Medicina Gen.mica y Toxicolog.a Ambiental, Instituto de Investigaciones Biom.dicas, Universidad Nacional Aut.noma de M.xico Ciudad Universitaria, Mexico City, Mexico. 130 Unidad de Biolog.a Molecular y Medicina Gen.mica, Instituto Nacional de Ciencias M.dicas y Nutrici.n, Mexico City, Mexico. 131 Division of Endocrine and Metabolism, Tri-Service General Hospital Songshan Branch, Taipei, Taiwan. 132 Shanghai Institute of Nutrition and Health, University of Chinese Academy of Sciences, Chinese Academy of Sciences, Shanghai, China. 133 Division of Genome Science, Department of Precision Medicine, National Institute of Health, Cheongju, Republic of Korea. 134 Biomedical Technology Research Center, Tokushima Research Institute, Otsuka Pharmaceutical Co., Tokushima, Japan. 135 Research Institute, National Center for Global Health and Medicine, Tokyo, Japan. 136 Department of Molecular Epidemiology, German Institute of Human Nutrition Potsdam-Rehbruecke, Nuthetal, Germany. 137 German Center for Diabetes Research (DZD), Neuherberg, Germany. 138 Department of Complex Trait Genetics, Center for Neurogenomics and Cognitive Research, Amsterdam Neuroscience, Vrije Universiteit Amsterdam, Amsterdam, The Netherlands. 139 Department of Child and Adolescent Psychiatry and Pediatric Psychology, Section Complex Trait Genetics, Amsterdam Neuroscience, Vrije Universiteit Medical Center, Amsterdam, The Netherlands. 140 Department of Biobank Research, Ume. University, Ume., Sweden. 141 Department of Odontology, Ume. University, Ume., Sweden. 142 Institute of Molecular and Clinical Ophthalmology Basel, Basel, Switzerland. 143 Privatpraxis Prof Jonas und Dr Panda-Jonas, Heidelberg, Germany. 144 Department of Ophthalmology, Medical Faculty Mannheim, Heidelberg University, Mannheim, Germany. 145 Beijing Institute of Ophthalmology, Beijing Tongren Eye Center, Beijing Tongren Hospital, Capital Medical University, Beijing Ophthalmology and Visual Sciences Key Laboratory, Beijing, China. 146 Center for Clinical Research and Prevention, Copenhagen University Hospital - Bispebjerg and Frederiksberg, Copenhagen, Denmark. 147 Department of Public Health, Faculty of Health and Medical Sciences, University of Copenhagen, Copenhagen, Denmark. 148 Faculty of Medicine, University of Kelaniya, Ragama, Sri Lanka. 149 Department of Geriatric and General Medicine, Osaka University Graduate School of Medicine, Suita, Japan. 150 Center for Genomic Medicine, Kyoto University Graduate School of Medicine, Kyoto, Japan. 151 Department of Psychiatry, University of Pennsylvania, Philadelphia, PA, USA. 152 Centre for Global Health, Usher Institute, University of Edinburgh, Edinburgh, UK. 153 Centre for Cardiovascular Sciences, Queens Medical Research Institute, University of Edinburgh, Edinburgh, UK. 154 Medical Research Institute, Kangbuk Samsung Hospital, Sungkyunkwan University School of Medicine, Seoul, Republic of Korea. 155 Department of Clinical Research Design and Evaluation (SAIHST), Sungkyunkwan University, Seoul, Republic of Korea. 156 SYNLAB MVZ Humangenetik Mannheim, Mannheim,

Germany. 157 Department of Clinical Sciences, Genetic and Molecular Epidemiology Unit, Lund University, Malm., Sweden. 158 Department of Computer Science, University of Toronto, Toronto, Ontario, Canada. 159 Department of Anthropology, University of Toronto at Mississauga, Mississauga, Ontario, Canada. 160 Genomics Research Centre, Centre for Genomics and Personalised Health, School of Biomedical Sciences, Queensland University of Technology, Kelvin Grove, Queensland, Australia. 161 Oneomics, Soonchunhyang Mirai Medical Center, Bucheon-si, Republic of Korea. 162 Laboratory of Neurogenetics, National Institute on Aging, National Institutes of Health, Bethesda, MD, USA. 163 Center for Alzheimer's and Related Dementias, National Institutes of Health, Bethesda, MD, USA. 164 Data Tecnica International, Glen Echo, MD, USA. 165 Cancer Genomics Research Laboratory, Leidos Biomedical Research, Rockville, MD, USA. 166 Department of Population and Quantitative Health Sciences, Case Western Reserve University, Cleveland, OH, USA. 167 Department of Medicine, University of Massachusetts Chan Medical School, Worcester, MA, USA. 168 Center for Geriatrics and Gerontology, Taichung Veterans General Hospital, Taichung, Taiwan. 169 Montreal Heart Institute, Montreal, Quebec, Canada. 170 Departments of Ophthalmology and Human Genetics, Radboud University Nijmegen Medical Center, Nijmegen, The Netherlands. 171 MRC Epidemiology Unit, University of Cambridge School of Clinical Medicine, Cambridge, UK. 172 Department of Clinical Science, Center for Diabetes Research, University of Bergen, Bergen, Norway. 173 Department of Clinical Sciences, Lund University Diabetes Centre, Malm., Sweden. 174 Department of Clinical Chemistry, Fimlab Laboratories, Tampere, Finland. 175 Department of Clinical Chemistry, Finnish Cardiovascular Research Center - Tampere, Faculty of Medicine and Health Technology, Tampere University, Tampere, Finland. 176 Department of Cardiology, Heart Center, Tampere University Hospital, Tampere, Finland. 177 National and Kapodistrian University of Athens, Dromokaiteio Psychiatric Hospital, Athens, Greece. 178 Department of Twin Research and Genetic Epidemiology, King's College London, London, UK. 179 NIHR Biomedical Research Centre at Guy's and St Thomas' Foundation Trust, London, UK. 180 Center for Public Health Genomics, University of Virginia School of Medicine, Charlottesville, VA, USA. 181 MRC Human Genetics Unit, Institute of Genetics and Cancer, University of Edinburgh, Western General Hospital, Edinburgh, UK. 182 Department of Psychiatry and Department of Community Health and Epidemiology, Dalhousie University, Halifax, Nova Scotia, Canada. 183 Institute of Psychiatric Phenomics and Genomics (IPPG), University Hospital, LMU Munich, Munich, Germany. 184 Institute for Medical Informatics, Biometry and Epidemiology, University Hospital Essen, Essen, Germany. 185 Diabetes Unit, Massachusetts General Hospital, Boston, MA, USA. 186 Center for Genomic Medicine, Massachusetts General Hospital, Boston, MA, USA. 187 Department of Psychiatry, Amsterdam Public Health and Amsterdam Neuroscience, Amsterdam UMC and Vrije Universiteit, Amsterdam, The Netherlands. 188 Biomedical and Translational Informatics Institute, Geisinger, Danville, PA, USA. 189 Department of Genetics, Institute for Biomedical Informatics, Perelman School of Medicine, University of Pennsylvania, Philadelphia, PA, USA. 190 MRC Population Health Research Unit, Nuffield Department of Population Health, University of Oxford, Oxford, UK. 191 Population Health Sciences, Bristol Medical School, University of Bristol, Bristol, UK. 192 Institute for Cardiogenetics, University of Lübeck, DZHK (German Research Centre for Cardiovascular Research) partner site Hamburg/Lübeck/Kiel and University Heart Center Lübeck, Lübeck, Germany. 193 Public Health Informatics Unit, Department of Integrated Health Sciences, Nagoya University Graduate School of Medicine, Nagoya, Japan. 194 Centre for Bone and Arthritis Research, Department of Internal Medicine and Clinical Nutrition, Institute of Medicine, Sahlgrenska Academy, University of Gothenburg, Gothenburg, Sweden. 195 Bioinformatics Core Facility, Sahlgrenska Academy, University of Gothenburg, Gothenburg, Sweden. 196 Korea Institute of Science and Technology, Gangneung Institute of Natural

Products, Gangneung, Republic of Korea. [197] Department of Clinical Biochemistry, Lillebaelt Hospital, Kolding, Denmark. [198] Department of Human Genetics, Wellcome Sanger Institute, Hinxton, UK. [199] Department of Internal Medicine, Section of Gerontology and Geriatrics, Leiden University Medical Center, Leiden, The Netherlands. [200] Institute of Genetics and Biophysics A. Buzzati-Traverso, CNR, Naples, Italy. [201] Department of Clinical Biochemistry and Immunology, Hospital of Southern Jutland, Aabenraa, Denmark. [202] The National Centre for Register-based Research, University of Aarhus, Aarhus, Denmark. [203] Centre for Population Health Research, University of Turku and Turku University Hospital, Turku, Finland. [204] Research Centre of Applied and Preventive Cardiovascular Medicine, University of Turku, Turku, Finland. [205] Medical School, University of Split, Split, Croatia. [206] Algebra University College, Zagreb, Croatia. [207] The Charles Bronfman Institute for Personalized Medicine, Icahn School of Medicine at Mount Sinai, New York, NY, USA. [208] Department of Environmental Medicine and Public Health, Icahn School of Medicine at Mount Sinai, New York, NY, USA. [209] Aragon Institute of Engineering Research, University of Zaragoza, Zaragoza, Spain. [210] Centro de Investigaci.n Biom.dica en Red en Bioingenier.a, Biomateriales y Nanomedicina (CIBER-BBN), Madrid, Spain. [211] Center for Non-Communicable Diseases, Karachi, Pakistan. [212] Department of Psychiatry, Interdisciplinary Center Psychopathology and Emotion Regulation, University of Groningen, University Medical Center Groningen, Groningen, The Netherlands. [213] Institute of Translational Genomics, Helmholtz Zentrum München, German Research Center for Environmental Health, Neuherberg, Germany. [214] Present address: Oxford Centre for Diabetes, Endocrinology and Metabolism, Radcliffe Department of Medicine, University of Oxford, Churchill Hospital, Oxford, UK. [215] Hunter Medical Research Institute, New Lambton Heights, New South Wales, Australia. [216] School of Medicine and Public Health, College of Health, Medicine and Wellbeing, The University of Newcastle, New Lambton Heights, New South Wales, Australia. [217] IRCCS Neuromed, Pozzilli, Italy. [218] Department of Medicine, Division of Endocrinology, Diabetes and Nutrition, University of Maryland School of Medicine, Baltimore, MD, USA. [219] Program for Personalized and Genomic Medicine, University of Maryland School of Medicine, Baltimore, MD, USA. [220] Unit of Genomics of Complex Diseases, Sant Pau Biomedical Research Institute (IIB Sant Pau), Barcelona, Spain. [221] Cardiovascular Medicine Unit, Department of Medicine, Karolinska Institutet, Center for Molecular Medicine, Stockholm, Sweden. [222] Laboratory of Epidemiology and Population Sciences, National Institute on Aging, National Institutes of Health, Baltimore, MD, USA. [223] Department of Biomedical Science, Hallym University, Chuncheon, Republic of Korea. [224] Istituto di Ricerca Genetica e Biomedica, Consiglio Nazionale delle Ricerche (CNR), Cagliari, Italy. [225] Department of Cell and Chemical Biology, Leiden University Medical Center, Leiden, The Netherlands. [226] Epidemiology and Data Science, Amsterdam UMC, location Vrije Universiteit Amsterdam, Amsterdam, The Netherlands. [227] Amsterdam Cardiovascular Sciences, Amsterdam, The Netherlands. [228] Department of Clinical Epidemiology, Leiden University Medical Center, Leiden, The Netherlands. [229] Icelandic Heart Association, K.pavogur, Iceland. [230] Wellcome Sanger Institute, Hinxton, UK. [231] deCODE Genetics/Amgen, Reykjavik, Iceland. [232] Mohn Nutrition Research Laboratory, Department of Clinical Science, University of Bergen, Bergen, Norway. [233] Department of Public Health Sciences, Parkinson School of Health Sciences and Public Health, Loyola University Chicago, Maywood, IL, USA. [234] VA Palo Alto Health Care System, Palo Alto, CA, USA. [235] Department of Medicine, Stanford University School of Medicine, Stanford, CA, USA. [236] Institute for Community Medicine, University Medicine Greifswald, Greifswald, Germany. [237] DZHK (German Centre for Cardiovascular Research), partner site Greifswald, Greifswald, Germany. [238] Cardiology Division, Department of Pediatrics, University of California, San Francisco, Oakland, CA, USA. [239] Centre for Cancer Genetic Epidemiology, University of Cambridge, Cambridge, UK. [240] Department of Public

Health and Primary Care, University of Cambridge, Cambridge, UK. [241] Department of Cardiology, Leiden University Medical Center, Leiden, The Netherlands. [242] Central Diagnostics Laboratory, Division Laboratories, Pharmacy and Biomedical Genetics, University Medical Center Utrecht, Utrecht University, Utrecht, The Netherlands. [243] Department of Human Genetics, Leiden University Medical Center, Leiden, The Netherlands. [244] Laboratory Genetic Metabolic Diseases, Department of Clinical Chemistry, Amsterdam UMC, University of Amsterdam, Amsterdam, The Netherlands. [245] Core Facility Metabolomics, Amsterdam UMC, University of Amsterdam, Amsterdam, The Netherlands. [246] Department of Cardiology, Division Heart and Lungs, University Medical Center Utrecht, Utrecht University, Utrecht, The Netherlands. [247] Department of Cardiology, University of Groningen, University Medical Center Groningen, Groningen, The Netherlands. [248] Department of Epidemiology and Biostatistics, School of Public Health, Tongji Medical College, Huazhong University of Science and Technology, Wuhan, China. [249] NIHR Barts Cardiovascular Biomedical Research Centre, Barts and The London School of Medicine and Dentistry, Queen Mary University of London, London, UK. [250] Department of Ophthalmology, Beijing Tongren Hospital, Capital Medical University, Beijing, China. [251] Department of Epidemiology and Biostatistics, MRC-PHE Centre for Environment and Health, School of Public Health, Imperial College London, London, UK. [252] Department of Dermatology, Medical University of Vienna, Vienna, Austria. [253] Department of Research and Innovation, Division of Clinical Neuroscience, Oslo University Hospital, Oslo, Norway. [254] Department of Neurology, Oslo University Hospital, Oslo, Norway. [255] MRC Unit for Lifelong Health and Ageing at UCL, Institute of Cardiovascular Science, University College London, London, UK. [256] Institute of Genetic Epidemiology, Faculty of Medicine and Medical Center, University of Freiburg, Freiburg, Germany. [257] Department of Medicine IV – Nephrology and Primary Care, Faculty of Medicine and Medical Center, University of Freiburg, Freiburg, Germany. [258] Institute of Molecular Medicine, McGovern Medical School, University of Texas Health Science Center at Houston, Houston, TX, USA. [259] Department of Medical Biochemistry, Kurume University School of Medicine, Kurume, Japan. [260] Rush Alzheimer's Disease Center, Rush University Medical Center, Chicago, IL, USA. [261] Department of Neurological Sciences, Rush University Medical Center, Chicago, IL, USA. [262] Department of Genetic Medicine, Weill Cornell Medicine-Qatar, Doha, Qatar. [263] Department of Computer and Systems Engineering, Alexandria University, Alexandria, Egypt. [264] Department of Cardiology, German Heart Centre Munich, Technical University Munich, Munich, Germany. [265] Department of Cardiology, Ealing Hospital, London North West University Healthcare NHS Trust, London, UK. [266] Department of Epidemiology and Biostatistics, Imperial College London, London, UK. [267] Cardiovascular Epidemiology Unit, Department of Public Health and Primary Care, University of Cambridge, Strangeways Research Laboratory, Cambridge, UK. [268] Department of Computational Medicine and Bioinformatics, University of Michigan, Ann Arbor, MI, USA. [269] Analytic and Translational Genetics Unit, Massachusetts General Hospital, Boston, MA, USA. [270] Stanley Center for Psychiatric Research, Broad Institute of MIT and Harvard, Cambridge, MA, USA. [271] Department of Nutrition, Gillings School of Global Public Health, University of North Carolina at Chapel Hill, Chapel Hill, NC, USA. [272] Carolina Population Center, University of North Carolina at Chapel Hill, Chapel Hill, NC, USA. [273] Department of Clinical Genetics, Erasmus MC, Rotterdam, The Netherlands. [274] Department of Radiology and Nuclear Medicine, Erasmus MC, Rotterdam, The Netherlands. [275] Latin American Brain Health (BrainLat), Universidad Adolfo Ib..ez, Santiago, Chile. [276] Unidad de Investigacion de Enfermedades Metabolicas and Direction of Nutrition, Instituto Nacional de Ciencias Medicas y Nutricion, Mexico City, Mexico. [277] Escuela de Medicina y Ciencias de la Salud, Tecnologico de Monterrey, Monterrey, Mexico. [278] Department of Epidemiology and Dean's Office, College of Public Health, University of Kentucky, Lexington, KY, USA. [279] Institute of Cardiovascular

Science, Faculty of Population Health Sciences, University College London, London, UK. 280 Health Data Research UK and Institute of Health Informatics, University College London, London, UK. 281 KG Jebsen Center for Genetic Epidemiology, Department of Public Health and Nursing, Faculty of Medicine and Health Sciences, Norwegian University of Science and Technology, NTNU, Trondheim, Norway. 282 HUNT Research Centre, Department of Public Health and Nursing, Norwegian University of Science and Technology, Levanger, Norway. 283 Department of Endocrinology, Clinic of Medicine, St. Olavs Hospital, Trondheim University Hospital, Trondheim, Norway. 284 Department of Nephrology, University Hospital Regensburg, Regensburg, Germany. 285 Geriatric Unit, Azienda Toscana Centro, Florence, Italy. 286 Systems Genomics Laboratory, School of Biotechnology, Jawaharlal Nehru University (JNU), New Delhi, India. 287 Institute of Molecular Genetics, National Research Council of Italy, Pavia, Italy. 288 Human Genetics Center and Department of Epidemiology, University of Texas Health Science Center at Houston, Houston, TX, USA. 289 Department of Nephrology and Rheumatology, Kliniken Südostbayern, Regensburg, Germany. 290 KfH Kidney Center Traunstein, Traunstein, Germany. 291 Center for Genomics and Personalized Medicine (CGPM), Aarhus University, Aarhus, Denmark. 292 Bioinformatics Research Centre, Aarhus University, Aarhus, Denmark. 293 USC-Office of Population Studies Foundation, University of San Carlos, Cebu City, Philippines. 294 Department of Nutrition and Dietetics, University of San Carlos, Cebu City, Philippines. 295 Human Genomics Laboratory, Pennington Biomedical Research Center, Baton Rouge, LA, USA. 296 Department of Biochemistry, Wake Forest School of Medicine, Medical Center Boulevard, Winston-Salem, NC, USA. 297 Department of Clinical Biochemistry, Lillebaelt Hospital, Vejle, Denmark. 298 Institute of Regional Health Research, University of Southern Denmark, Odense, Denmark. 299 Clinic of Medicine, St. Olavs Hospital, Trondheim University Hospital, Trondheim, Norway. 300 Lee Kong Chian School of Medicine, Nanyang Technological University, Singapore, Singapore. 301 Imperial College Healthcare NHS Trust, Imperial College London, London, UK. 302 Adjunct Faculty, JSS University Academy of Higher Education and Research (JSSAHER), JSS (Deemed to be) University, Mysuru, India. 303 Ophthalmology and Visual Sciences Academic Clinical Program (Eye ACP), Duke-NUS Medical School, Singapore, Singapore. 304 Department of Medical Genetics, Oslo University Hospital, Oslo, Norway. 305 Department of Medical Research, B.rum Hospital, Vestre Viken Hospital Trust, Gjettum, Norway. 306 Department of Neurology, Division of Vascular Neurology, University of Maryland School of Medicine, Baltimore, MD, USA. 307 Baltimore Veterans Affairs Medical Center, Department of Neurology, Baltimore, MD, USA. 308 Unidad de Investigaci.n M.dica en Bioqu.mica, Hospital de Especialidades, Centro M.dico Nacional Siglo XXI, Instituto Mexicano del Seguro Social, Mexico City, Mexico. 309 Dipartimento di Scienze Biomediche, Universit. degli Studi di Sassari, Sassari, Italy. 310 Intermountain Heart Institute, Intermountain Medical Center, Murray, UT, USA. 311 Department of Surgery, University of Pennsylvania, Philadelphia, PA, USA. 312 Corporal Michael J. Crescenz VA Medical Center, Philadelphia, PA, USA. 313 Department of Vascular Surgery, University Medical Center Utrecht, University of Utrecht, Utrecht, The Netherlands. 314 Department of Human Nutrition, Wageningen University, Wageningen, The Netherlands. 315 Center for Translational and Computational Neuroimmunology, Department of Neurology, Columbia University Medical Center, New York, NY, USA. 316 Department of Oncology, University of Cambridge, Cambridge, UK. 317 Department of General Practice, Amsterdam Public Health Institute, Amsterdam UMC, location VUmc, Amsterdam, The Netherlands. 318 Department of Nutrition, Harvard T.H. Chan School of Public Health, Boston, MA, USA. 319 Cardiac Arrhythmia Service, Massachusetts General Hospital, Boston, MA, USA. 320 Cardiovascular Research Center, Massachusetts General Hospital, Boston, MA, USA. 321 Department of Clinical Sciences in Malm., Division of Geriatric Medicine, Lund University, Malm., Sweden. 322 Molecular Cardiology Division, Victor

Chang Cardiac Research Institute, Darlinghurst, New South Wales, Australia. [323] Cardiology Department, St Vincent's Hospital, Darlinghurst, New South Wales, Australia. [324] Faculty of Medicine, UNSW Sydney, Kensington, New South Wales, Australia. [325] Translational Gerontology Branch, National Institute on Aging, National Institutes of Health, Baltimore, MD, USA. [326] Robertson Center for Biostatistics, University of Glasgow, Glasgow, UK. [327] Human Genetics Center, School of Public Health, University of Texas Health Science Center at Houston, Houston, TX, USA. [328] Department of Public Health and Clinical Medicine, Umeå University, Umeå, Sweden. [329] Department of Internal Medicine, Wake Forest School of Medicine, Medical Center Boulevard, Winston-Salem, NC, USA. [330] Faculty of Veterinary and Agricultural Science, University of Melbourne, Parkville, Victoria, Australia. [331] Agriculture Victoria Research, Department of Jobs, Precincts and Regions, Bundoora, Victoria, Australia. [332] Injury Prevention Research Center, University of North Carolina at Chapel Hill, Chapel Hill, NC, USA. [333] Division of Physical Therapy, University of North Carolina at Chapel Hill, Chapel Hill, NC, USA. [334] Centro de Investigacion en Salud Poblacional Instituto Nacional de Salud Publica and Centro de Estudios en Diabetes, Cuernavaca, Mexico. [335] Division of Human Genetics, Children's Hospital of Philadelphia, Philadelphia, PA, USA. [336] Departments of Pediatrics and Genetics, Perelman School of Medicine, University of Pennsylvania, Philadelphia, PA, USA. [337] Division of Endocrinology and Diabetes, Children's Hospital of Philadelphia, Philadelphia, PA, USA. [338] Faculty of Medicine, University of Iceland, Reykjavik, Iceland. [339] Department of Preventive Medicine, Keck School of Medicine of USC, Los Angeles, CA, USA. [340] Department of Pediatrics, Perelman School of Medicine, University of Pennsylvania, Philadelphia, PA, USA. [341] Division of Pulmonary Medicine, Children's Hospital of Philadelphia, Philadelphia, PA, USA. [342] Institute of Biomedical and Clinical Science, University of Exeter Medical School, Exeter, UK. [343] Cardiovascular Health Research Unit, Department of Epidemiology, University of Washington, Seattle, WA, USA. [344] Department of Paediatrics, Yong Loo Lin School of Medicine, National University of Singapore, Singapore, Singapore. [345] Khoo Teck Puat - National University Children's Medical Institute, National University Health System, Singapore, Singapore. [346] Department of Internal Medicine II, Division of Cardiology, Medical University of Vienna, Vienna, Austria. [347] Menzies Research Institute Tasmania, University of Tasmania, Hobart, Tasmania, Australia. [348] Centre for Eye Research Australia, Royal Victorian Eye and Ear Hospital, University of Melbourne, Melbourne, Victoria, Australia. [349] Lions Eye Institute, Centre for Ophthalmology and Vision Science, University of Western Australia, Perth, Western Australia, Australia. [350] Cardiology Division, Massachusetts General Hospital, Boston, MA, USA. [351] Department of Genetics, Shanghai-MOST Key Laboratory of Heath and Disease Genomics, Chinese National Human Genome Center and Shanghai Industrial Technology Institute, Shanghai, China. [352] Department of Internal Medicine, University of Utah, Salt Lake City, UT, USA. [353] Australian Centre for Precision Health, Clinical and Health Sciences, University of South Australia, Adelaide, South Australia, Australia. [354] South Australian Health and Medical Research Institute, Adelaide, South Australia, Australia. [355] Department of Environmental and Preventive Medicine, Jichi Medical University School of Medicine, Shimotsuke, Japan. [356] Department of Epidemiology and Population Health, Albert Einstein College of Medicine, Bronx, NY, USA. [357] Division of Endocrinology, Diabetes and Metabolism, School of Medicine, Ohio State University, Columbus, OH, USA. [358] Center for Life Course Health Research, Faculty of Medicine, University of Oulu, Oulu, Finland. [359] Unit of Primary Health Care, Oulu University Hospital, OYS, Oulu, Finland. [360] Department of Life Sciences, College of Health and Life Sciences, Brunel University London, Uxbridge, UK. [361] The Eye Hospital, School of Ophthalmology and Optometry, Wenzhou Medical University, Wenzhou, China. [362] Einthoven Laboratory for Experimental Vascular Medicine, LUMC, Leiden, The Netherlands. [363] Netherlands Heart Institute, Utrecht,

The Netherlands. 364 Department of Clinical Physiology, Tampere University Hospital, Tampere, Finland. 365 Department of Clinical Physiology, Finnish Cardiovascular Research Center - Tampere, Faculty of Medicine and Health Technology, Tampere University, Tampere, Finland. 366 Laboratory of Complex Trait Genomics, Department of Computational Biology and Medical Sciences, Graduate School of Frontier Sciences, The University of Tokyo, Tokyo, Japan. 367 Department of Ophthalmology, The Catholic University of Korea Incheon St. Mary's Hospital, Incheon, Republic of Korea. 368 NIHR Oxford Biomedical Research Centre, Churchill Hospital, Oxford, UK. 369 German Centre for Cardiovascular Research (DZHK), partner site Munich Heart Alliance, Munich, Germany. 370 Radboud University Medical Center, Radboud Institute for Health Sciences, Department of Urology, Nijmegen, The Netherlands. 371 Corneal Dystrophy Research Institute, Yonsei University College of Medicine, Seoul, Republic of Korea. 372 Saevit Eye Hospital, Goyang, Republic of Korea. 373 Department of Biochemistry, College of Medicine, Ewha Womans University, Seoul, Republic of Korea. 374 Department of Cardiology, University Heart and Vascular Center UKE Hamburg, Hamburg, Germany. 375 Institute of Cardiovascular Sciences, College of Medical and Dental Sciences, University of Birmingham, Birmingham, UK. 376 German Center for Cardiovascular Research, partner site Hamburg/Kiel/Lübeck, Hamburg, Germany. 377 Atrial Fibrillation NETwork, Münster, Germany. 378 Department of Epidemiology and Public Health, UCL Institute of Epidemiology and Health Care, University College London, London, UK. 379 Healthy Longevity Translational Research Programme, Yong Loo Lin School of Medicine, National University of Singapore, Singapore, Singapore. 380 University of Helsinki and Department of Medicine, Helsinki University Hospital, Helsinki, Finland. 381 Minerva Foundation Institute for Medical Research, Helsinki, Finland. 382 Department of Preventive Cardiology, Lipoprotein Apheresis Unit and Lipid Disorders Clinic, Metropolitan Hospital, Athens, Greece. 383 MRC-PHE Centre for Environment and Health, Imperial College London, London, UK. 384 National Heart and Lung Institute, Imperial College London, London, UK. 385 Medical Department III – Endocrinology, Nephrology, Rheumatology, University of Leipzig Medical Center, Leipzig, Germany. 386 Institute for Social and Economic Research, University of Essex, Colchester, UK. 387 Institute of Clinical Medicine, Internal Medicine, University of Eastern Finland and Kuopio University Hospital, Kuopio, Finland. 388 Department of Medicine, University of Colorado at Denver, Aurora, CO, USA. 389 Berlin Institute of Health at Charit. – Universit.tsmedizin Berlin, Berlin, Germany. 390 Epidemiology Program, University of Hawaii Cancer Center, Honolulu, HI, USA. 391 Department of Internal Medicine, Ewha Womans University School of Medicine, Seoul, Republic of Korea. 392 Department of Epidemiology and Biostatistics, Peking University Health Science Center, Beijing, China. 393 Peking University Center for Public Health and Epidemic Preparedness and Response, Beijing, China. 394 Institute of Epidemiology and Biobank Popgen, Kiel University, Kiel, Germany. 395 Key Laboratory of Systems Health Science of Zhejiang Province, Hangzhou Institute for Advanced Study, University of Chinese Academy of Sciences, Chinese Academy of Sciences, Hangzhou, China. 396 Department of Clinical Medicine, Faculty of Health and Medical Sciences, University of Copenhagen, Copenhagen, Denmark. 397 Division of Cardiovascular Medicine and Abboud Cardiovascular Research Center, University of Iowa Hospitals and Clinics, Iowa City, IA, USA. 398 Alliance for Human Development, Lunenfeld-Tanenbaum Research Institute, Sinai Health System, Toronto, Ontario, Canada. 399 Department of Medical Epidemiology and Biostatistics, Karolinska Institutet, Stockholm, Sweden. 400 Division of Cardiology, University of California, San Francisco, San Francisco, CA, USA. 401 Department of Medicine, Internal Medicine, Lausanne University Hospital, Lausanne, Switzerland. 402 University of Lausanne, Lausanne, Switzerland. 403 SYNLAB Academy, SYNLAB Holding Deutschland, Mannheim, Germany. 404 Clinical Institute of Medical and Chemical Laboratory Diagnostics, Medical University of Graz, Graz, Austria.

405 Department of Medicine, Division of Cardiology, Duke University School of Medicine, Durham, NC, USA. 406 Duke Molecular Physiology Institute, Duke University School of Medicine, Durham, NC, USA. 407 Psychiatric Genetics, QIMR Berghofer Medical Research Institute, Brisbane, Queensland, Australia. 408 Geriatric Medicine, Institute of Medicine, Sahlgrenska Academy, University of Gothenburg, Gothenburg, Sweden. 409 Geriatrics Research and Education Clinical Center, Baltimore Veterans Administration Medical Center, Baltimore, MD, USA. 410 Centre for Vision Research and Department of Ophthalmology, Westmead Millennium Institute of Medical Research, University of Sydney, Sydney, New South Wales, Australia. 411 Department of Public Health and Primary Care, Leiden University Medical Center, Leiden, The Netherlands. 412 Usher Institute of Population Health Sciences and Informatics, University of Edinburgh, Edinburgh, UK. 413 Electrophysiology Section, Division of Cardiovascular Medicine, Perelman School of Medicine, University of Pennsylvania, Philadelphia, PA, USA. 414 Department of Medicine, University of North Carolina at Chapel Hill, Chapel Hill, NC, USA. 415 Department of Chronic Diseases, Norwegian Institute of Public Health, Oslo, Norway. 416 Department of Pain Management and Research, Oslo University Hospital, Oslo, Norway. 417 Institute of Human Genetics, School of Medicine and University Hospital Bonn, Bonn, Germany. 418 Sahlgrenska University Hospital, Department of Drug Treatment, Gothenburg, Sweden. 419 Laboratorio de Inmunogen.mica y Enfermedades Metab.licas, Instituto Nacional de Medicina Gen.mica, CDMX, Mexico City, Mexico. 420 Paavo Nurmi Centre, Sports and Exercise Medicine Unit, Department of Physical Activity and Health, University of Turku, Turku, Finland. 421 Institute for Precision Health, David Geffen School of Medicine at UCLA, Los Angeles, CA, USA. 422 Pat MacPherson Centre for Pharmacogenetics and Pharmacogenomics, Division of Population Health and Genomics, School of Medicine, University of Dundee, Ninewells Hospital and Medical School, Dundee, UK. 423 Centre Nutrition, Sant. et Soci.t. (NUTRISS), Institute of Nutrition and Functional Foods, Universit. Laval, Qu.bec City, Quebec, Canada. 424 IBE-Chair of Epidemiology, LMU Munich, Neuherberg, Germany. 425 Population, Policy and Practice, UCL Great Ormond Street Hospital Institute of Child Health, London, UK. 426 Department of Medicine, University of Pennsylvania, Philadelphia, PA, USA. 427 Department of Clinical Physiology and Nuclear Medicine, Turku University Hospital, Turku, Finland. 428 Hero DMC Heart Institute, Dyanand Medical College, Ludhiana, India. 429 Second Department of Cardiology, Medical School, National and Kapodistrian University of Athens, University General Hospital Attikon, Athens, Greece. 430 Division of Biostatistics, Washington University School of Medicine, St Louis, MO, USA. 431 Genetics, Merck Sharp & Dohme, Kenilworth, NJ, USA. 432 Department of Epidemiology, University of Washington, Seattle, WA, USA. 433 Department of Endocrinology and Metabolism, Kyung Hee University School of Medicine, Seoul, Korea. 434 Department of Public Health, Clinicum, Faculty of Medicine, University of Helsinki, Helsinki, Finland. 435 Departments of Medicine, Pharmacology, and Biomedical Informatics, Vanderbilt University Medical Center, Nashville, TN, USA. 436 Department of Epidemiology and Data Science, Amsterdam Public Health Institute, Amsterdam Cardiovascular Sciences Institute, Amsterdam UMC, location VUmc, Amsterdam, The Netherlands. 437 Department of Cardiology and Department of Medicine, Columbia University, New York, NY, USA. 438 Department of Pediatrics, Section of Genetics, College of Medicine, University of Oklahoma Health Sciences Center, Oklahoma City, OK, USA. 439 Department of Pharmaceutical Sciences, University of Oklahoma Health Sciences Center, Oklahoma City, OK, USA. 440 Department of Physiology, University of Oklahoma Health Sciences Center, Oklahoma City, OK, USA. 441 Oklahoma Center for Neuroscience, University of Oklahoma Health Sciences Center, Oklahoma City, OK, USA. 442 Institute of Cardiovascular and Medical Sciences, University of Glasgow, Glasgow, UK. 443 Gottfried Schatz Research Center (for Cell Signaling, Metabolism and Aging), Medical

University of Graz, Graz, Austria. 444 Institute of Nutritional Science, University of Potsdam, Nuthetal, Germany. 445 Deutsches Herzzentrum München, Cardiology, Deutsches Zentrum für Herz- und Kreislaufforschung (DZHK) – Munich Heart Alliance, and Technische Universit.t München, München, Germany. 446 School of Biomedical Science and Pharmacy, University of Newcastle, New Lambton Heights, New South Wales, Australia. 447 Department of Medicine, Vanderbilt University Medical Center, Nashville, TN, USA. 448 Central University of Punjab, Bathinda, India. 449 Department of Medicine I, University Hospital, LMU Munich, Munich, Germany. 450 Department of Cardiology, Clinical Sciences, Lund University and Sk.ne University Hospital, Lund, Sweden. 451 The Wallenberg Laboratory, Department of Molecular and Clinical Medicine, Institute of Medicine, Gothenburg University and the Department of Cardiology, Sahlgrenska University Hospital, Gothenburg, Sweden. 452 Wallenberg Center for Molecular Medicine and Lund University Diabetes Center, Lund University, Lund, Sweden. 453 Population Health Research Institute, St George’s, University of London, London, UK. 454 Molecular Epidemiology Section, Department of Biomedical Data Sciences, Leiden University Medical Center, Leiden, The Netherlands. 455 Department of Genetics, Stanford University School of Medicine, Stanford, CA, USA. 456 Department of Medicine, Faculty of Medicine, Universit. de Montr.al, Montreal, Quebec, Canada. 457 Helsinki University Central Hospital, Research Program for Clinical and Molecular Metabolism, University of Helsinki, Helsinki, Finland. 458 Folkh.lsan Research Center, Helsinki, Finland. 459 Department of Public Health, University of Helsinki, Helsinki, Finland. 460 Diabetes Research Group, King Abdulaziz University, Jeddah, Saudi Arabia. 461 Unidad de Biolog.a Molecular y Medicina Gen.mica, Instituto de Investigaciones Biom.dicas, UNAM, Mexico City, Mexico. 462 Instituto Nacional de Ciencias M.dicas y Nutrici.n Salvador Zubir.n, Mexico City, Mexico. 463 Yong Loo Lin School of Medicine, National University of Singapore and National University Health System, Singapore, Singapore. 464 Milken Institute School of Public Health, The George Washington University, Washington, DC, USA. 465 Department Geriatric Medicine, Amsterdam Public Health, Amsterdam UMC location University of Amsterdam, Amsterdam, The Netherlands. 466 Department of Epidemiology and Data Science, Amsterdam UMC, Amsterdam, The Netherlands. 467 Interfaculty Institute for Genetics and Functional Genomics, University Medicine Greifswald, Greifswald, Germany. 468 Unidad de Investigaci.n M.dica en Epidemiolog.a Cl.nica, Hospital de Especialidades, Centro M.dico Nacional Siglo XXI, Instituto Mexicano del Seguro Social, Mexico City, Mexico. 469 Institute of Cellular Medicine, Newcastle University, Newcastle upon Tyne, UK. 470 Department of Population and Public Health Sciences, Keck School of Medicine of USC, Los Angeles, CA, USA. 471 Department of Physiology and Neuroscience, Keck School of Medicine of USC, Los Angeles, CA, USA. 472 USC Diabetes and Obesity Research Institute, Keck School of Medicine of USC, Los Angeles, CA, USA. 473 Lundbeck Foundation Center for GeoGenetics, GLOBE Institute, University of Copenhagen, Copenhagen, Denmark. 474 School of Chinese Medicine, China Medical University, Taichung, Taiwan. 475 Diabetes Unit, KEM Hospital and Research Centre, Pune, India. 476 Kurume University School of Medicine, Kurume, Japan. 477 Division of Cancer Control and Population Sciences, UPMC Hillman Cancer Center, University of Pittsburgh, Pittsburgh, PA, USA. 478 Department of Epidemiology, Graduate School of Public Health, University of Pittsburgh, Pittsburgh, PA, USA. 479 TUM School of Medicine, Technical University of Munich and Klinikum Rechts der Isar, Munich, Germany. 480 Institute of Clinical Medicine, Faculty of Medicine, University of Oslo, Oslo, Norway. 481 Department of Population Health Sciences, Geisinger, Danville, PA, USA. 482 Vanderbilt Genetics Institute, Division of Genetic Medicine, Vanderbilt University Medical Center, Nashville, TN, USA. 483 Department of Medicine, Veterans Affairs Boston Healthcare System, Boston, MA, USA. 484 Department of Epidemiology, Emory University Rollins School of Public Health, Atlanta, GA, USA. 485 Atlanta VA Health Care System, Decatur, GA, USA.

486 Princess Al-Jawhara Al-Brahim Centre of Excellence in Research of Hereditary Disorders (PACER-HD), King Abdulaziz University, Jeddah, Saudi Arabia. 487 The Mindich Child Health and Development Institute, Icahn School of Medicine at Mount Sinai, New York, NY, USA. 488 School of Life Sciences, Westlake University, Hangzhou, China. 489 Westlake Laboratory of Life Sciences and Biomedicine, Hangzhou, China. 490 Department of Human Genetics, University of Michigan, Ann Arbor, MI, USA. 491 McDonnell Genome Institute and Department of Medicine, Washington University School of Medicine, St Louis, MO, USA. 492 Laboratory of Statistical Immunology, Immunology Frontier Research Center (WPI-IFReC), Osaka, Japan. 493 Integrated Frontier Research for Medical Science Division, Institute for Open and Transdisciplinary Research Initiatives, Osaka University, Osaka, Japan. 494 Programs in Metabolism and Medical and Population Genetics, Broad Institute of MIT and Harvard, Cambridge, MA, USA. 495 Departments of Pediatrics and Genetics, Harvard Medical School, Boston, MA, USA. 496 Present address: Department of Mathematics and Statistics, St Cloud State University, St Cloud, MN, USA. 497 Genentech, South San Francisco, CA, USA. 498 Present address: Laboratory for Systems Genetics, RIKEN Center for Integrative Medical Sciences, Kanagawa, Japan. 499 Department of Genome Informatics, Graduate School of Medicine, The University of Tokyo, Tokyo, Japan.

The All of Us Research Program Consortia

Brian Ahmedani[1]; Christine D Cole Johnson[1]; Habib Ahsan[2]; Hoda Anton-Culver[4]; Eric Topol[5]; Katie Baca-Motes[5]; Julia Moore-Vogel[5]; Praduman Jain[6]; Mark Begale[6]; Neeta Jain[6]; David Klein, MBA[6]; Scott Sutherland[6]; Bruce Korf[7]; Beth Lewis[7]; Ali G Gharavi[8]; George Hripcsak[8]; Eric Boerwinkle[9]; Scott Joseph Hebbring[10]; Elizabeth Burnside[11]; Dorothy Farrar-Edwards[11]; Amy Taylor[12]; Liliana Lombardi Desa[13]; Steve Thibodeau[14]; Mine Cicek[14]; Eric Schlueter[15]; Beverly Wilson Holmes[15]; Martha Daviglus[16]; Paul Harris[+17]; Consuelo Wilkins[17]; Dan Roden[17]; Kim Doheny[18]; Evan Eichler[19]; Gail Jarvik[19]; Gretchen Funk[20]; Anthony Philippakis[21]; Heidi Rehm[21]; Stacey Gabriel[21]; Richard Gibbs[22]; Edgar M Gil Rico[23]; David Glazer[24]; Jessica Burke[25]; Philip Greenland[26]; Elizabeth Shenkman[27]; William R Hogan[27]; Priscilla Igho-Pemu[28]; Elizabeth W Karlson[29]; Jordan Smoller[29]; Shawn N Murphy[29]; Margaret Elizabeth Ross[30]; Rainu Kaushal[30]; Eboni Winford[31]; Vik Kheterpal[32]; Francisco A Moreno[33]; Cheryl Thomas[34]; Mitchell Lunn[35]; Juno Obedin-Maliver[35]; Oscar Marroquin[36]; Shyam Visweswaran[36]; Steven Reis[36]; Patrick McGovern[37]; Gregory Talavera[38]; George T O'Connor[39]; Lucila Ohno-Machado[41]; Fornessa Randal[43]; Andreas A Theodorou[44]; Eric Reiman[44]; Mercedita Roxas-Murray[45]; Louisa Stark[46]; Ronnie Tepp[47]; Alicia Zhou[48]; Scott Topper[48]; Rhonda Trousdale[49]; Phil Tsao[50]; Scott T Weiss[51]; Jeffrey Whittle[53]; Stephan Zuchner[55]; Olveen Carrasquillo[55]; Megan Lewis[57]; Jen Uhrig[57]; May Okihiro[58]; Maria Argos[16]; Brisa Aschebook-Kilfoy[16]; Laura Bartlett[54]; Roberta Carlin[59]; Elizabeth Cohn[60]; Vivian Colon-Lopez[61]; Karl Cooper[59]; Linda Cottler[62]; Errol Crook[63]; Elizabeth Culler[64]; Charles Drum[59]; Milton Eder[62]; Mark Edmunds[52]; Rachel Everhart[65]; Adolph Falcon[23]; Becky Fein[66]; Zeno Frano[53]; Michael Garrett[67]; Sandra Halverson[68]; Eileen Handberg[27]; Joyce Ho[26]; Laura Horne[66]; Rosario Isasi[55]; Jessica Isom[69]; Jessica Jarmin[70]; Megan Jula[71]; Royan Kamyar[72]; Frida Kleiman[60]; Isaac Kohane[73]; Babbette Lamarca[67]; Brendan Lee[22]; Niall Lennon[21]; Dessie Levy[74]; Todd Mahr[75]; Emily Makahi[58]; Vivienne Marshall[76];Elizabeth Mayer-Davis[77]; Jacob McCauley[55]; Jeffrey McKinney[78]; David McPherson[9]; Robert Meller[28]; Jose Melo[61]; David Ming-Hung Lin[79]; Michael Minor[74]; Evan Muse[5]; Kapil Parakh[80]; Cathryn Peltz-Rauchman[1]; Linda Perez Laras[81]; Subhara Raveendran[82]; Gail Reilly[31]; Jody Reilly[83]; Nelida Rivera[81]; Laura Rosales[22]; Tracie Rosser[56]; Linda Salgin[38]; Sherilyn Sawyer[84]; William Simonson[85]; Amy Sitapati[41]; Cynthia So-Armah[69]; Gene Stegeman[86]; Christin Suver[87]; Michael Taitel[42]; Kyla Taylor[31]; Daniel Hernandez Tinoco[31]; Scott Topper[48]; Rhonda Trousdale[49]; Jason Vassy[84]; Jamie Walz[78]; Preston Watkins[88]; Blaker Wilkerson[89]; Katrina Yamazaki[12]; Melissa Basford[17]; Amaryllis Silva Boschetti[41]; Matthew Breeden[90]; Suchitra Chandrasekaran[56]; Cheryl Clark[51]; Kim Enard[90]; Yuri Fresko[83]; Richard Grucza[90]; Robert Kelley[56];

Kathleen Keogh[13]; Monica Kraft[91]; Christopher Lough[92]; Ted Malmstrom[90]; Paul Nemeskal[69]; Matt Pagel[56]; Jeffrey Scherrer[90]; Sanjay Skukla[10]; Debra Smith[93]; Bryce Turner[94]; Miriam Vos[56];

Affiliations
1. Henry Ford Health System
2. University of Chicago Medical Center
3. Jackson-Hinds Comprehensive Health Center
4. University of California, Irvine
5. Scripps Research Translational Institute
6. Vibrent Health
7. University of Alabama at Birmingham
8. Columbia University
9. University of Texas Health Science Center at Houston
10. Marshfield Clinic Research Institute
11. University of Wisconsin at Madison
12. Community Health Center, Inc.
13. Sun River Health
14. Mayo Clinic and Foundation, Rochester
15. Cooperative Health
16. University of Illinois at Chicago
17. Vanderbilt University Medical Center
18. Johns Hopkins University School of Medicine
19. University of Washington
20. FiftyForward
21. Broad Institute
22. Baylor College of Medicine
23. National Alliance for Hispanic Health
24. Verily Life Sciences
25. MITRE Corporation
26. Northwestern University
27. University of Florida
28. Morehouse School of Medicine, Atlanta
29. Partners Health Care
30. Cornell University, Weill Medical College
31. Cherokee Health Systems
32. CareEvolution, Inc.
33. University of Arizona, Tucson
34. Delta Research and Educational Foundation
35. Stanford University
36. University of Pittsburgh
37. Wondros
38. San Ysidro Health Center
39. Boston Medical Center
40. VA All of Us Coordinating Center, Boston
41. University of California, San Diego
42. Walgreen Co.
43. Asian Health Coalition
44. Banner Health

45. Montage Marketing Group
46. University of Utah
47. HCM Strategists
48. Color Genomics, Inc.
49. NYC Health + Hospitals
50. VA AoU Coordinating Center - Palo Alto
51. Brigham and Women's Hospital
52. San Diego Blood Bank
53. Medical College of Wisconsin
54. National Library of Medicine (NLM)
55. University of Miami School of Medicine
56. Emory University
57. Research Triangle Institute
58. Waianae Coast CHC
59. American Association of Health and Disability
60. Hunter College
61. University of Puerto Rico Comprehensive Cancer Center
62. CTSA Community Engagement Programs
63. University of South Alabama
64. TPC: Blood Assurance
65. TPC: Denver Health
66. TPC: Active Minds
67. University of Mississippi Medical Center
68. TPC: DLH Corp
69. Mass General Hospital
70. Tactis
71. TPC: Mary’s Center
72. TPC: Owaves
73. Harvard Medical School
74. National Baptist Convention
75. Gundersen Health System
76. South Texas Blood and Tissue Center
77. University of North Carolina at Chapel Hill
78. Sensis
79. TPC: Bloodworks Northwest
80. TPC: Fitbit
81. COSSMA
82. Patients Like Me
83. Quest Diagnostics Incorporated
84. VA AoU Coordinating Center
85. Cascade Regional BLood Services
86. ExamOne
87. Sage Bionetworks
88. WebMD Health Corp
89. Blue Cross Blue Shield
90. Saint Louis University
91. Mount Sinai Health System
92. LifeSouth
93. SunCoast Blood Center

94. University Southern California
