# Supplementary material for: Selection, optimization and validation of ten chronic disease polygenic risk scores for clinical implementation in diverse US populations
Source: Nat Med. 2024 Feb 19;30(2):480–7. doi: 10.1038/s41591-024-02796-z (PMC10878968; doi:10.1038/s41591-024-02796-z)
Supplement: Supplementary file 2 — Reporting Summary [file 41591_2024_2796_MOESM2_ESM.pdf]

nature portfolio

Corresponding author(s): Niall lennon
Last updated by author(s): Dec 6 2023

Reporting Summary

Nature Portfolio wishes to improve the reproducibility of the work that we publish. This form provides structure for consistency and transparency in reporting. For further information on Nature Portfolio policies, see our Editorial Policies and the Editorial Policy Checklist.

Statistics

For all statistical analyses, confirm that the following items are present in the figure legend, table legend, main text, or Methods section.

n/a	Confirmed	
☐	☒	The exact sample size (*n*) for each experimental group/condition, given as a discrete number and unit of measurement
☐	☒	A statement on whether measurements were taken from distinct samples or whether the same sample was measured repeatedly
☐	☒	The statistical test(s) used AND whether they are one- or two-sided<br>*Only common tests should be described solely by name; describe more complex techniques in the Methods section.*
☐	☒	A description of all covariates tested
☐	☒	A description of any assumptions or corrections, such as tests of normality and adjustment for multiple comparisons
☐	☒	A full description of the statistical parameters including central tendency (e.g. means) or other basic estimates (e.g. regression coefficient) AND variation (e.g. standard deviation) or associated estimates of uncertainty (e.g. confidence intervals)
☐	☒	For null hypothesis testing, the test statistic (e.g. *F*, *t*, *r*) with confidence intervals, effect sizes, degrees of freedom and *P* value noted<br>*Give P values as exact values whenever suitable.*
☒	☐	For Bayesian analysis, information on the choice of priors and Markov chain Monte Carlo settings
☒	☐	For hierarchical and complex designs, identification of the appropriate level for tests and full reporting of outcomes
☐	☒	Estimates of effect sizes (e.g. Cohen's *d*, Pearson's *r*), indicating how they were calculated

Our web collection on statistics for biologists contains articles on many of the points above.

Software and code

Policy information about availability of computer code

Data collection	Data collection for participants (age, sex-at-birth, ancestry) was collected in a Redcap software (v14.0.1) hosted by Vanderbilt University.
Data analysis	Imputation and PRS pipeline code is made available in github and a link has been added to the online methods section.

For manuscripts utilizing custom algorithms or software that are central to the research but not yet described in published literature, software must be made available to editors and reviewers. We strongly encourage code deposition in a community repository (e.g. GitHub). See the Nature Portfolio guidelines for submitting code & software for further information.

Data

Policy information about availability of data

All manuscripts must include a data availability statement. This statement should provide the following information, where applicable:

- Accession codes, unique identifiers, or web links for publicly available datasets
- A description of any restrictions on data availability
- For clinical datasets or third party data, please ensure that the statement adheres to our policy

Underlying data used to validate performance of PRS pipeline are available in dbGAP https://emerge-network.org/dbgap/
De-identified data relating to trial participants will be available through dbGaP (https://www.ncbi.nlm.nih.gov/gap/) access and the AnVIL platform (https://anvil.terra.bio/) as an interim analysis in 2024 and final dataset at the end of the study, expected in 2026.
Information (sites and weights) on the implemented scores can be found at https://github.com/broadinstitute/eMERGE-implemented-PRS-models-Lennon-et-al and also on the UCSC browser https://genome.ucsc.edu/s/Max/emerge
Additionally, PGS Catalog IDs for most of the implemented scores are indicated in Supplementary Table 3.

Human research participants

Policy information about studies involving human research participants and Sex and Gender in Research.

Reporting on sex and gender	Sex-at-birth information is captured at enrollment and used in the triaging of certain PRS results (e.g. Breast Cancer PRS is only calculated for participants declaring sex-at-birth of female; Prostate Cancer PRS is only calculated for participants declaring sex-at-birth of male).
Population characteristics	Age is a relevant covariate as described in the manuscript as some scores are returned only for adult participants, some for pediatric only, and some for both. Sex-at-birth is also a relevant covariate as described above. Self-reported ancestry is also captured at enrollment but is not used in the PRS calculation, rather computed genetic ancestry is used.
Recruitment	The eMERGE network aims to increase applicability of genomic risk prediction across populations by validating PRS in multiple ancestral groups and enrolling a prospective cohort that includes individuals who are currently underrepresented in clinical-genomic research. Six sites are committed to recruiting an “enhanced diversity cohort” with a target of 75% of individuals belonging to a racial or ethnic minority or medically underserved population, whereas the remainder of clinical sites will target 35%. Enrollment is not targeted to individuals with specific conditions, although individuals with prevalent conditions can be included. The network focuses on 3 major aims: (1) recruit 25,000 individuals (ages 3-75 years) from general health care system populations, (2) generate cross-ancestry and ancestry-adjusted PRS as the basis for reports to return risk alongside family health history, clinical, and monogenic risk, and (3) measure individual outcomes and provider behaviors and comprehension in response to receiving this information.
Ethics oversight	The Vanderbilt University Medical Center CC is the institutional review board of record (#211043) for the network’s single institutional review board, approved in July 2021.

Note that full information on the approval of the study protocol must also be provided in the manuscript.

Field-specific reporting

Please select the one below that is the best fit for your research. If you are not sure, read the appropriate sections before making your selection.

☒ Life sciences ☐ Behavioural & social sciences ☐ Ecological, evolutionary & environmental sciences

For a reference copy of the document with all sections, see nature.com/documents/nr-reporting-summary-flat.pdf

Life sciences study design

All studies must disclose on these points even when the disclosure is negative.

Sample size	The expected numbers of participants that would meet study thresholds for “high risk GIRA” were calculated based on the genetic datasets used to develop and test the PRS, as described in the manuscript.
Data exclusions	No data were excluded
Replication	Within the validation phase the imputation and PRS pipelines were successfully assessed for repeatability and reproducibility as described in the Methods.
Randomization	The first 2,500 subjects analyzed represent each of the first 2,500 individuals recruited into this study. The order of subject analysis was random
Blinding	The analysts were blinded to actual diagnoses at time of PRS calculation.

Reporting for specific materials, systems and methods

We require information from authors about some types of materials, experimental systems and methods used in many studies. Here, indicate whether each material, system or method listed is relevant to your study. If you are not sure if a list item applies to your research, read the appropriate section before selecting a response.

Materials & experimental systems

n/a	Involved in the study
☒	☐ Antibodies
☒	☐ Eukaryotic cell lines
☒	☐ Palaeontology and archaeology
☒	☐ Animals and other organisms
☒	☐ Clinical data
☒	☐ Dual use research of concern

Methods

n/a	Involved in the study
☒	☐ ChIP-seq
☒	☐ Flow cytometry
☒	☐ MRI-based neuroimaging

Eukaryotic cell lines

Policy information about cell lines and Sex and Gender in Research

Cell line source(s)	
Authentication	
Mycoplasma contamination	
Commonly misidentified lines (See ICLAC register)	
